# siRNA Off-Target Effects Can Be Reduced at Concentrations That Match Their Individual Potency

**DOI:** 10.1371/journal.pone.0021503

**Published:** 2011-07-05

**Authors:** Daniel R. Caffrey, Juan Zhao, Zhili Song, Michael E. Schaffer, Steven A. Haney, Romesh R. Subramanian, Albert B. Seymour, Jason D. Hughes

**Affiliations:** 1 Pfizer Inc, Cambridge, Massachusetts, United States of America; 2 Centocor R&D Inc., Radnor, Pennsylvania, United States of America; 3 Research and Development, Shire HGT, Lexington, Massachusetts, United States of America; 4 Merck Research Laboratories, Boston, Massachusetts, United States of America; Victor Chang Cardiac Research Institute (VCCRI), Australia

## Abstract

Small interfering RNAs (siRNAs) are routinely used to reduce mRNA levels for a specific gene with the goal of studying its function. Several studies have demonstrated that siRNAs are not always specific and can have many off-target effects. The 3′ UTRs of off-target mRNAs are often enriched in sequences that are complementary to the seed-region of the siRNA. We demonstrate that siRNA off-targets can be significantly reduced when cells are treated with a dose of siRNA that is relatively low (e.g. 1 nM), but sufficient to effectively silence the intended target. The reduction in off-targets was demonstrated for both modified and unmodified siRNAs that targeted either STAT3 or hexokinase II. Low concentrations reduced silencing of transcripts with complementarity to the seed region of the siRNA. Similarly, off-targets that were not complementary to the siRNA were reduced at lower doses, including up-regulated genes that are involved in immune response. Importantly, the unintended induction of caspase activity following treatment with a siRNA that targeted hexokinase II was also shown to be a concentration-dependent off-target effect. We conclude that off-targets and their related phenotypic effects can be reduced for certain siRNA that potently silence their intended target at low concentrations.

## Introduction

RNA interference (RNAi) is a natural gene silencing process employed by both plants and animals. Laboratory methods using the mechanism have been devised to allow for knockdown of the mRNA levels of nearly any gene of interest. The technology is commonly employed in gene-specific and genome-wide functional assays [1] and offers the potential to develop a novel class of therapeutics [2]. Two of the major types of RNAi include small interfering RNA (siRNA) and microRNA (miRNA) [3].

In mammals, siRNA are typically 21 nucleotides in length and consist of a guide strand and a complementary passenger strand. The siRNA is bound by the RNA-induced silencing complex (RISC), which facilitates the cleavage and/or degradation of mRNA that are complementary to the guide strand [4]. These short double-stranded RNA molecules are typically produced by the Dicer enzyme, which cleaves both exogenous and endogenous double stranded RNA [5], [6], [7]. However, Dicer-mediated processing can be bypassed by transfecting mammalian cells with ∼21 nucleotide double-stranded RNA. The duplex typically consists of 19 complementary nucleotides with 2- nucleotide overhangs at the 3′ end.

miRNA are non-coding RNA genes that are expressed in both plants and animals. The initial transcripts go through various processing steps, including the generation of ∼80 nucleotide stem loop pre-miRNA and Dicer cleavage to produce a mature 22 nucleotide double stranded RNA [8], [9], [10], [11]. The mature miRNA is bound by the RISC complex, which in turn, binds to the 3′UTRs of mRNAs and results in mRNA degradation and/or translation repression. In miRNA, the seed region that typically spans nucleotides 2-7 is complementary to the 3′UTR target sites and is primarily responsible for translation repression [12]. The rest of the guide strand is partially complementary to the 3′UTR and appears to be less important for mRNA silencing.

Clearly, there are a number of similarities between the miRNA pathway and the siRNA pathway. Indeed, siRNAs can function as miRNAs [13], [14] and several studies have shown that siRNA can regulate unintended transcripts via seed complementarity in their 3′UTRs [7], [15], [16], [17], [18], [19]. Such off-targets can produce false positives in siRNA screens [20] and have the potential to cause undesired side effects in a clinical trial. The human transcriptome contains thousands of 3′UTR stretches that are complementary to any given seed region, and it is difficult to determine which of these seed-matches are capable of being *bona fide* miRNA-like off-targets. While seed accessibility is clearly an important parameter for miRNA-like binding [21], [22], we have not succeeded in using accessibility-based methods to accurately predict miRNA off-targets in human cells. Encouragingly, a recent study demonstrated that it is possible to reduce miRNA-like off-targets by introducing a 2′-O-methyl ribosyl substitution at position 2 of the guide strand [23]. Furthermore, 2′-O-methyl modifications of the passenger strand can eliminate the immunostimulatory effects that are sometimes associated with siRNA [24], [25].

In theory, the partial base-pairing between a siRNA and a 3′UTR is expected to be less thermodynamically stable than a full-length siRNA-mRNA duplex. We therefore hypothesized that siRNA with high affinity for their intended target could be used at a minimum effective dose such that miRNA-like off-targets would be significantly reduced or eliminated. We tested this hypothesis with both modified and unmodified siRNA and the results are described below.

## Results

### Potent siRNA

**Figure 1:**
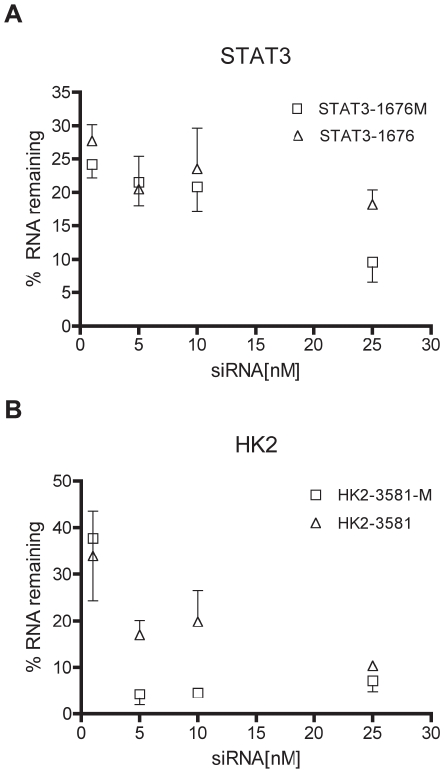
siRNAs potently silence their intended targets. RT-PCR of purified RNA demonstrated that all concentrations of siRNA reduced mRNA levels of the intended targets by 50% or more relative to control. A) STAT3-1676 and STAT3-1676M. B) HK2-3581 and HK2-3581M.

To study off-target effects, we designed siRNAs that targeted hexokinase II (HK2) and STAT3 and had minimal complementarity with other transcripts encoded by the human genome (Table 1, see Materials and Methods). To determine whether a minimum effective dose could reduce miRNA-like off-targets, it was important to use potent siRNA. Using RT-PCR, both STAT3-1676 and HK2-3581 were shown to reduce mRNA levels of their respective targets by 50% or more at 1 nM (Figure 1A). Both STAT3-1676M and HK2-3581-M, which contain a 2′-O-methyl modification at position 2, also silenced their respective targets at levels that were similar to the unmodified siRNA (Figure 1B). Therefore, these siRNA were considered sufficiently potent to test our hypothesis.

**Table 1:** siRNA sequences used in this study.

siRNA	Guide strand	Passenger strand
AllStars negative control	UUUGUAAUCGUCGAUACCC	GGGUAUCGACGAUUACAAA
HK2-3581/HK2-3581M	UUGUUGUGCAUCUCCACUCuu	GAGUGGAGAUGCACAACAAuu
HK2-4031	UCCAUGUUCACACACAUCCuu	GGAUGUGUGUGAACAUGGAuu
PLK1	Pool	Pool
siGenome2 non-targeting control	GUAUCUCUUCAUAGCCUUA	UAAGGCUAUGAAGAGAUAC
STAT3-1676/STAT3-1676M	UUGGUCAGCAUGUUGUACCuu	GGUACAACAUGCUGACCAAuu

The canonical seed region, positions 2–7, is underlined in the guide strand. 3′ overhangs are in lowercase. The modified (M) versions are identical in sequence to the unmodified siRNA and contain a 2′-O-methyl ribosyl substitution at position 2 of the guide strand.

### Definition of off-targets

To assess off-targets, cells were treated with each siRNA at 1 nM, 10 nM, and 25 nM and compared to non-targeting control siRNA using Affymetrix microarrays. Off-targets were defined as transcripts with a 2-fold change in mRNA level (RMA normalized values) and a P-value less than or equal to 0.05 at any dose. This definition of off-targets allows for the inclusion of both up-regulated and down-regulated transcripts that are not necessarily bound by the siRNA/RISC complex.

### Off-targets for unmodified siRNA are reduced at minimum effective doses

**Figure 2:**
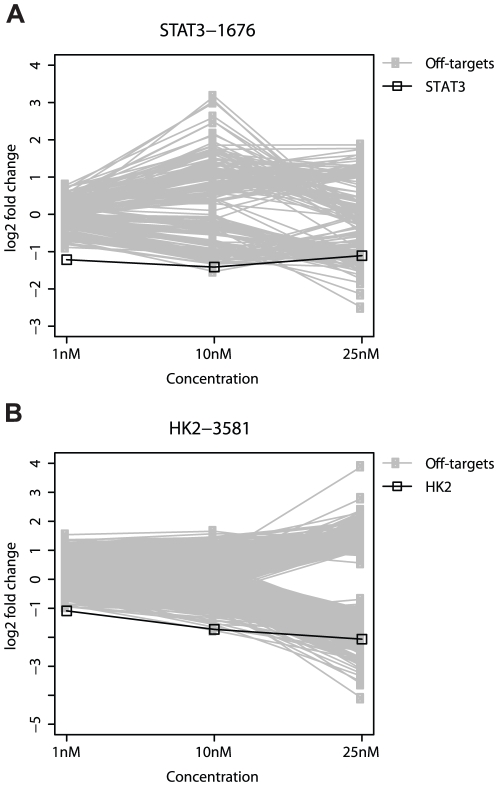
Off-targets for unmodified siRNA. A) Cells were transfected with the specified doses of STAT3-1676. B) Cells were transfected with the specified doses of HK2-3581. The greatest changes in off-target transcript levels were observed at the higher concentrations. The intended target was the most significantly down-regulated transcript at 1 nM.

Using the above definition of off-targets, we observed a total of 174 off-targets (2 fold change at any dose) when MCF-7 cells were treated with STAT3-1676. Off-targets for STAT3-1676 (Figures 2A and S1) are described in detail below.

At 25 nM, there were 38 off-targets that were down-regulated more than STAT3 (Figure 2A, S1, and Table 2). Consistent with previous findings, the majority of these down-regulated off-targets possess a stretch of 3′UTR that is complementary to the seed region (32/56). In contrast, the majority of the up-regulated off-targets do not possess a 3′UTR region that is complementary to siRNA seed region. Interestingly, 9 of the genes with at least a 2-fold increase in expression were annotated as immune response genes (Table S1). The set of up-regulated off-targets was significantly enriched in the ‘immune response' GO term when compared to all other genes in the genome (P = 1.6e-13, Table S2).

**Table 2:** Summary of off-targets.

siRNA	Log_2_FC target	↓ > target	2-fold ↓ (seed)	2-fold ↑(seed)
STAT3-1676 (25 nM)	−2.2	38	56(32)	54(7)
STAT3-1676 (10 nM)	−2.7	1	30(22)	63(11)
STAT3-1676 (1 nM)	−2.3	0	0(0)	0(0)
STAT3-1676M (25 nM)	−3.8	5	102(65)	219(48)
STAT3-1676M (10 nM)	−3.8	1	23(20)	30(6)
STAT3-1676M (1 nM)	−3.7	0	1(1)	0(0)
HK2-3581 (25 nM)	−4.2	77	728(288)	430(4)
HK2-3581 (10 nM)	−3.3	1	42(24)	89(0)
HK2-3581 (1 nM)	−2.1	0	0(0)	41(0)
HK2-3581M (25 nM)	−2.5	66	202(73)	67(6)
HK2-3581M (10 nM)	−2.6	0	0(0)	0(0)
HK2-3581M (1 nM)	−1.47	1	0(0)	0(0)

The table shows the log_2_ fold change for the intended target, the number of transcripts that are down-regulated more than the intended target (↓ >target), the number of transcripts that are down-regulated 2-fold or more along with the subset that are complementary to seed region 2–7 (2-fold ↓ (seed)), and the number of transcripts that are up-regulated by 2-fold or more along with the subset that are complementary to seed region 2–7 ((2-fold ↑ (seed)).

Decreasing the siRNA concentration from 25 nM to 10 nM did not have a significant effect on STAT3 silencing (Figure 2A, S1, and Table 2), but the number of off-targets was reduced such that only 1 gene (DDR1) was down-regulated more than STAT3. The number of off-targets with a 2-fold decrease in mRNA levels was decreased from 56 transcripts (25 nM) to 30 transcripts (10 nM). Again, the majority of these down-regulated off-targets (22/30) have a site in their 3′UTR that is 100% complementary to the seed region of the siRNA. The immune response genes continued to be up-regulated.

When the siRNA concentration was reduced to 1 nM, there was still a more than 2-fold decrease in STAT3 mRNA levels, which was not significantly different than the levels observed at the higher siRNA doses (Figure 2A, S1, and Table 2). Importantly, STAT3 was the most significantly knocked-down transcript at 1 nM and none of the off-targets that were observed at the higher doses were down-regulated by two-fold or more. Indeed, there were only 6 off-targets with more than a 1.6-fold decrease in mRNA levels at 1 nM, whereas there were 67 off-targets with this level of down-regulation at 25 nM. All of the immune response genes that were up-regulated at the higher doses were reduced to a 1.6 fold change or less at 1 nM, suggesting that the apparent immune response was independent of STAT3 silencing.

For HK2-3581, we observed 1169 off-targets that were significantly up- or down-regulated at one or more doses in Hep3B cells. Off-targets for HK2-3581 (Figures 2B and S2) are described in detail below.

At 25 nM, we observed a 4.2 fold decrease in HK2 mRNA levels and there were 77 off-targets that were silenced at greater levels than HK2 (Figure 2B, S2, and Table 2). Of the 728 off-targets that exhibited more than a 2-fold decrease in mRNA levels, 288 possess a stretch of 3′UTR that is complementary to the seed region. In contrast, only 4 of the 430 off-targets with a 2-fold increase in mRNA levels have a 3′UTR that is complementary to the seed region of the siRNA. Interestingly, the majority of off-targets that were annotated as being involved in immune response were down-regulated (Table S3).

When the concentration of HK2-3581 was reduced from 25 nM to 10 nM, there was a 3.3 fold decrease in HK2 mRNA levels compared to the 4.2 fold decrease observed at 25 nM (Figure 2B, S2, and Table 2). However, the decrease in siRNA concentration had a greater effect on the number of off-targets. The number of off-targets that were down-regulated more strongly than HK2 was reduced from 77 to 1. Nevertheless, there were still 42 off-targets with a 2-fold decrease in mRNA levels and 24 of these had a 3′UTR that was complementary to the seed region. Furthermore, there were 89 off-targets with a 2-fold increase in mRNA levels at 10 nM.

When the siRNA concentration was reduced to 1 nM, there was a 2.1-fold decrease in HK2 mRNA levels compared to the 3.3-fold decrease observed at 10 nM (Figure 2B, S2, and Table 2), but HK2 was the most potently down-regulated gene and all of the off-targets that were observed at the higher concentrations exhibited less than a 2-fold decrease in mRNA levels. Interestingly, there were still 41 off-targets with a 2-fold increase in mRNA levels at 1 nM.

Taken together, the above results demonstrate that it is possible to identify a minimum effective dose for certain siRNA, such that potential off-targets are significantly reduced. At these lower doses, the intended targets were the most potently down-regulated genes. For STAT3, the lower dose did not significantly decrease the level of silencing. Although a decrease in HK2 silencing was observed at 1 nM, there was still a 2-fold decrease in mRNA levels. Importantly, our qPCR experiments demonstrated that mRNA levels for both STAT3 and HK2 were decreased by more than 60% at the lower 1 nM concentration (Figure 1).

Furthermore, off-targets that lacked seed complementarity or were annotated as being involved in immune response were also significantly reduced at the lowest dose.

### Modified siRNA have fewer off-targets at minimum effective doses

Previous studies have demonstrated that off-targets can be reduced by including a 2′-O-methyl ribosyl substitution at position 2 of the guide strand [23]. To confirm these observations and to determine whether off-targets could be further reduced at minimum effective doses, we incorporated this modification into STAT3-1676-M and HK2-3581-M (Table 1). In q-PCR experiments, both STAT3-1676M and HK2-3581M were shown to silence their respective targets at levels that were similar to their unmodified versions (Figure 1).

**Figure 3:**
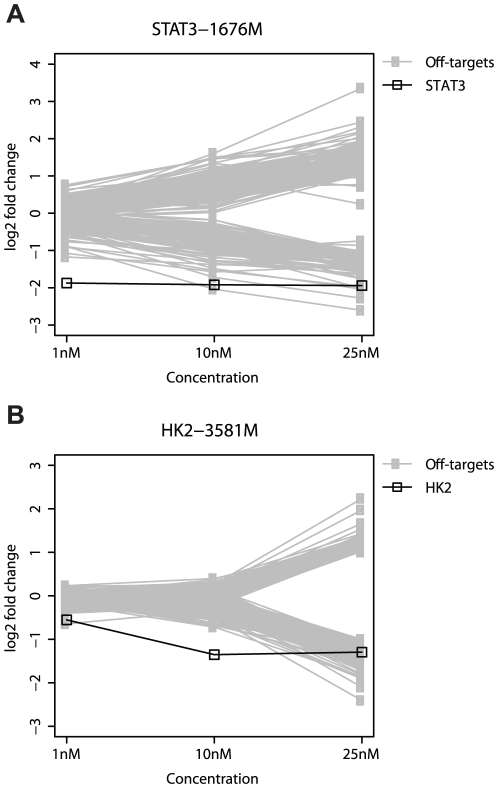
Off-targets for modified siRNA. A) Cells were transfected with the specified doses of STAT3-1676M. B) Cells were transfected with the specified doses of HK2-3581M. The greatest changes in off-target transcript levels were observed at the higher concentrations. STAT3 was the most significantly down-regulated transcript at 1 nM. HK2 was the most significantly down-regulated transcript at10 nM.

Using the previously described criteria for defining off-targets, we detected 325 off-targets for STAT3-1676M that were either up or down-regulated (Figures 3A and S3). Surprisingly, this was significantly more than the 174 off-targets observed for the unmodified STAT3-1676 and demonstrates that this particular modification does not always reduce the number of off-targets. The differences between STAT3-1676M and STAT3-1676 were primarily observed at the highest concentration (Table 2) and are further described below.

At 25 nM, there was a 3.8 fold decrease in STAT3 mRNA levels, which was more than the 2.2 fold decrease observed for the unmodified STAT3-1676. This difference is probably due to biological or experimental variation as the unmodified siRNA was slightly more potent in the qPCR experiments. Due to the relatively potent silencing of STAT3 by STAT3-1676M, there were only 5 off-targets that were down-regulated more than STAT3 (Figures 3A, S3, and Table 2). However, there were 102 off-targets with a 2-fold decrease in mRNA levels and 65 of these off-targets possess a 3′UTR that is complementary to the seed region of STAT3-1676M. In contrast, the same concentration of unmodified STAT3-1676 caused a 2-fold decrease in 56 off-targets where 32 of them were complementary to the seed region in their 3′UTRs. There were 219 off-targets with a two-fold increase in expression compared to 54 off-targets observed for the unmodified STAT3-1676. Similar to the unmodified siRNA, there were 9 off-targets with a two-fold increase in expression that were also annotated as being involved in immune response.

When the concentration of STAT3-1676M was decreased from 25 nM to 10 nM, there was no change in the level of STAT3 silencing (Figure 3A, S3, and Table 2). However, the number of down-regulated off-targets was significantly decreased at 10 nM and was slightly less than the number observed for unmodified STAT3-1676 at the same concentration. The majority of off-targets (20/23) that were down-regulated 2-fold or more have a 3′UTR that is complementary to the seed region of STAT3-1676M. The number of off-targets that were up-regulated by two-fold or more was reduced to 30 genes compared to the 63 genes observed for the unmodified STAT3-1676. Only 2 genes from this set of these up-regulated off-targets are annotated as being involved in immune response.

Reducing the concentrations of STAT3-1676M from 10 nM to 1 nM did not significantly alter STAT3 silencing but did reduce off-targets further (Figure 3A, S3, and Table 2). There was only one off-target that was down-regulated by two-fold or more and STAT3 was clearly the most down-regulated gene. Similar to the unmodified STAT3-1676, there were no off-targets that were up-regulated by two-fold or more and the up-regulation of genes involved in immune response was mitigated.

For HK2-3581M, there were 270 off-targets (up or down-regulated) detected at one or more doses, which was significantly less than the 1169 off-targets observed for the unmodified HK2-3581M (Figures 3B and S4).

At 25 nM, 66 off-targets were down-regulated at greater levels than HK2 which was slightly fewer than the 77 off-targets down-regulated at greater levels than HK2 in response to the unmodified HK2-3581 (Figure 3B, S4, and Table 2). However, the number of off-targets that were down-regulated 2-fold or more was considerably fewer for HK2-3581M (202 off-targets) than for HK2-3581 (728 off-targets). Consistent with this, the number of off-targets with seed-complementarity to the 3′UTR was also less for HK2-3581M (73 off-targets) than for HK2-3581 (288 off-targets). The number of off-targets that were up-regulated by two-fold or more was also reduced for HK2-3581M (67 off-targets) relative to HK2-3581 (430 off-targets).

Despite the ability of the 2′-O-methyl ribosyl modification to significantly reduce off-targets, it was clear that modification of this particular siRNA was not sufficient to eliminate all off-targets at this dose.

Reducing the dose of HK2-3581M from 25 nM to 10 nM dramatically reduced the number of off-targets without having an effect on HK2 silencing (Figures 3B, S4, and Table 2). There was a 2.6-fold silencing of HK2 and none of the off-targets were down-regulated more than the intended target. Whereas unmodified HK2-3581 altered the expression of several genes by two-fold or more, HK2-3581M did not induce a two-fold expression of any off-targets at 10 nM.

When the concentration of HK2-3581M was reduced from 10 nM to 1 nM, silencing of HK2 was decreased from 2.6 fold to 1.47 fold (Figure 3B, S4, and Table 2). Indeed, one off-target (DSG2) was down-regulated slightly more (1.5-fold decrease) than the intended target. Once again, there were no off-targets that were down or up-regulated by two-fold or more, compared to unmodified HK2-3581, which induced a two-fold increase in expression of 41 genes at the same concentration. As HK2 silencing was also reduced at 1 nM, the optimal dose is probably between 1 nM and 10 nM.

The above results confirm that 2′-O-methyl ribosyl modifications can effectively reduce off-targets for some but not all siRNA, and demonstrate that the reduction in off-targets can extend beyond 3′UTRs that are complementary to the seed region. Importantly, we demonstrate that a low effective dose can significantly reduce off-targets that still exist for a modified siRNA at higher doses.

### 3′UTR off-targets

**Figure 4:**
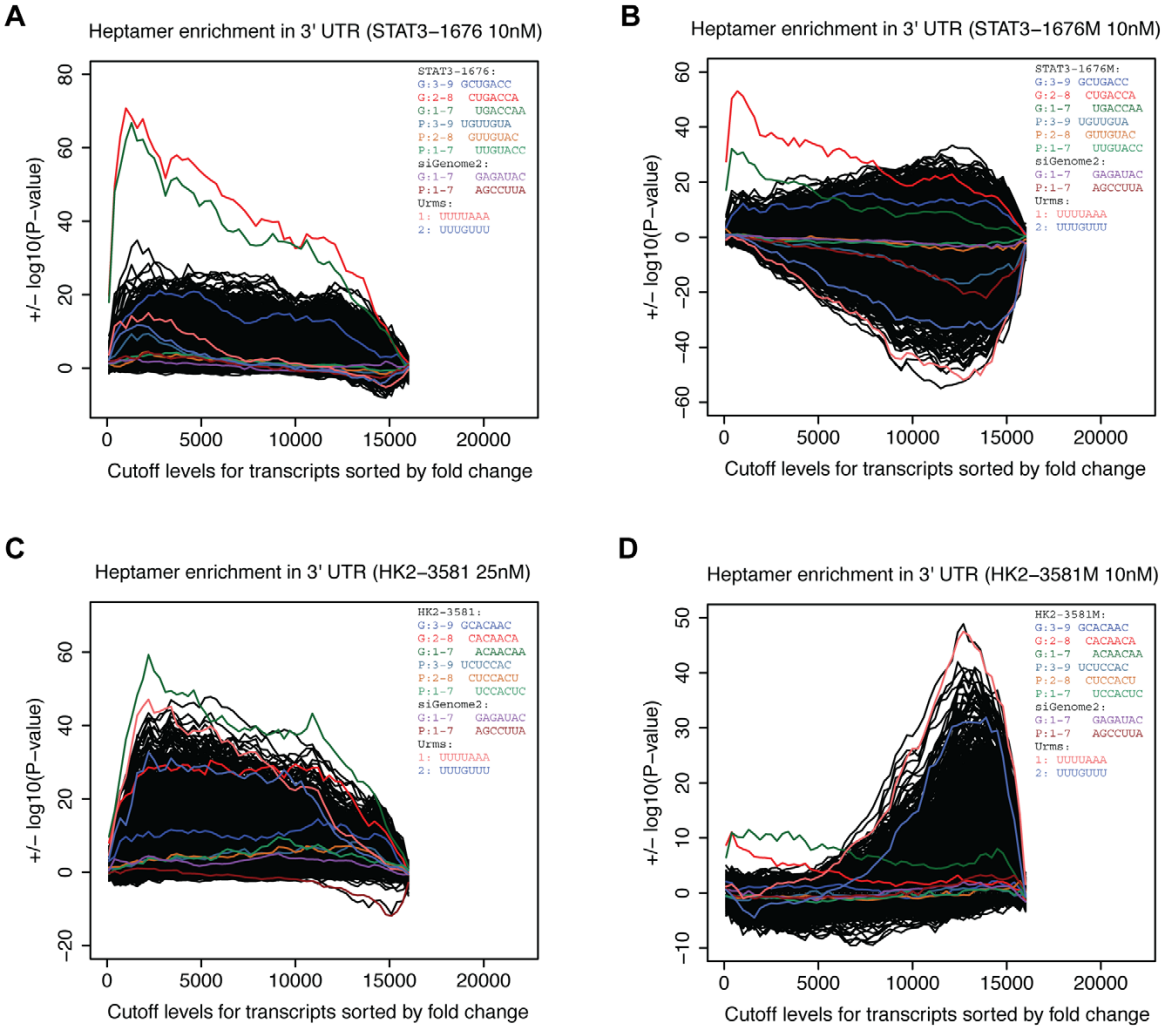
3′UTR heptamer enrichment analysis for each siRNA. A) Enrichment of 3′UTRS with seed complementarity to STAT3-1676 was most striking at 10 nM. B) Enrichment of 3′UTRS with seed complementary to STAT3-1676M was most striking at 10 nM. C) Enrichment of 3′UTRS with seed complementary to HK2-3581 was most striking at 25 nM. D) Enrichment of 3′UTRS with seed complementary to HK2-3581M was most striking at 10 nM. Transcripts from each microarray were rank-ordered by log_2_ fold-change and P-values were computed at different levels of fold-change (increments of 100). The hyper-geometric test was used to assess whether a particular heptamer was over or under-represented in 3′UTRs at each level of fold-change.

To further evaluate the role of seed complementarity in the 3′UTR of off-targets at different concentrations, we analyzed each microarray experiment with an implementation of the Sylamer method [26]. Using a series of fold-change cut-off levels, corresponding 3′UTRs were grouped as being above or below each fold-change threshold. For each grouping, hypergeometric tests were performed to determine whether one set of 3′UTRs were significantly over or under-represented in a particular k-mer (4^k^ possible k-mers) relative to the other set of 3′UTRs. As an exhaustive analysis of all k-mers is performed, we can determine if a k-mer of interest is over or under-represented relative to all other control k-mers. The analysis was performed for both hexamers and heptamers at all concentrations (Figures S5-12) and concentrations where heptamers are particularly enriched in the 3′UTRs of off-targets are shown in Figure 4.

Although we previously observed more off-targets with seed complementarity in their 3′UTRs at 25 nM than the lower concentrations (Table 2 and Figures S1-4), enrichment of off-targets with seed complementarity was generally more striking at 10 nM than 25 nM (Figures S5–12). This is because off-targets that are not complementary to the seed region were also increased at 25 nM.

Clearly, off-targets for both STAT3-1676 and STAT3-1676M were over-represented in 3′UTRs that are complementary to the seed regions of the siRNA (Figure 4A and 4B). Among the initial cut-offs, where down-regulated transcripts were compared to other transcripts, the most significantly enriched heptamers (CUGACCA and UGACCAA) are complementary to seed region 2–8 and 1–7 of the siRNA respectively. The passenger strands for STAT3-1676 and STAT3-1676M did not have a major effect on mRNA levels, as the P-values for the complementary heptamers (UGUUGUA, GUUGUAC, and UUGUACC) were among background P-values. Similarly, heptamers that are complementary to the seed region of the passenger and guide strands of the non-targeting control were among background P-values. For example, heptamers that are complementary to positions 1–7 of the passenger and guide strands (AGCCUUA and GAGAUAC) are among background P-values (Figure 4A and 4B).

Figure 4C shows that down-regulated off-targets for HK2-3581(25 nM) were over-represented in 3′UTR heptamers that are complementary to the seed region. In particular, the down-regulated off-targets had 3′UTRs that were enriched in the heptamer ACAACAA, which is complementary to seed region 1–7. For HK2-3581M, the heptamers (ACAACAA and CACAACA), which are complementary to seed regions 1–7 and 2–8, were the most over-represented heptamers among down-regulated transcripts (Figure 4D). The passenger strands for HK2-3581 and HK2-3581M did not have a major impact on mRNA levels as the complementary heptamers (UCUCCAC, CUCCACU, UCCACUC) were among background P-values. Again, heptamers that are complementary to positions 1–7 of the non-targeting control (passenger and guide strands) were among background P-values for down-regulated transcripts.

Recently, the U-rich motifs URM1 (UUUUAAA) and URM2 (UUUGUUU) which are bound by the ELAV4 RNA binding protein [27] were shown to be enriched in the 3′UTRS of transcripts down-regulated by miRNA and siRNA [28]. For both HK2-3581 and HK2-3581-M, URM1 is among the most enriched heptamers at various fold-change cut-off levels (Figure 4C-D, S10 and S12).

For STAT3-1676-M (Figure 4B and S8), URM1 is under-represented at the higher concentrations, particularly among the up-regulated transcripts. This under-representation among up-regulated transcripts was unexpected and may indicate that the non-targeting control siRNA tends to bind 3′UTRs that contain URM1. For the unmodified STAT3-1676 (Figure 4A and S6), URM1 is among the over-represented heptamers found in 3′UTRs that belong to down-regulated transcripts that were detected at 25 nM and 1 nM. The URM2 motif was not over or under-represented among any of the off-targets that were detected in response to our siRNA (Figures 4, S6, S8, S10, and S12). Indeed, the URM2 motif was among the background P-values that were observed for the control heptamers. Although the URM1 and URM2 motifs were previously shown to be over-represented in the 3′UTRs of down-regulated transcripts, the results for our siRNA were less conclusive.

Collectively, the above experiments demonstrated that down-regulated off-targets are enriched in 3′UTRs that are complementary to seed region of both modified siRNA and unmodified siRNA. The 2′-O-methyl ribosyl modification at position 2 of the guide strand is designed to make seed-3′UTR interactions less favorable and does indeed reduce the number of off-targets for HK2 (Table 2). Nevertheless, the k-mer enrichment analysis demonstrates that seed-3′UTR complementarity can still be an issue for modified siRNA, particularly at higher doses.

### HK2-3581 induces caspase activity

As described above, we observed 1155 off-targets for HK2-3581, suggesting a potential for significant phenotypic effects at higher doses. The majority of down-regulated off-targets possessed 3′UTRs that were not complementary to the seed region (Table 2). Up-regulated transcripts for HK2-3581 were significantly enriched in GO terms relating to cell cycle and a slightly less striking enrichment was also observed for HK2-3581M (Tables S4–S7).

Furthermore, visual inspection of the cells following treatment with HK2-3581, suggested that the cells were undergoing apoptosis. To confirm this, we compared HK2-3581 to HK2-4031 in caspase activation and cell proliferation assays. We chose HK2-4031 as it had significantly fewer off-targets than HK2-3581 and consistent with our previous findings, its off-targets were also reduced at a lower concentration (Figure S13).

**Figure 5:**
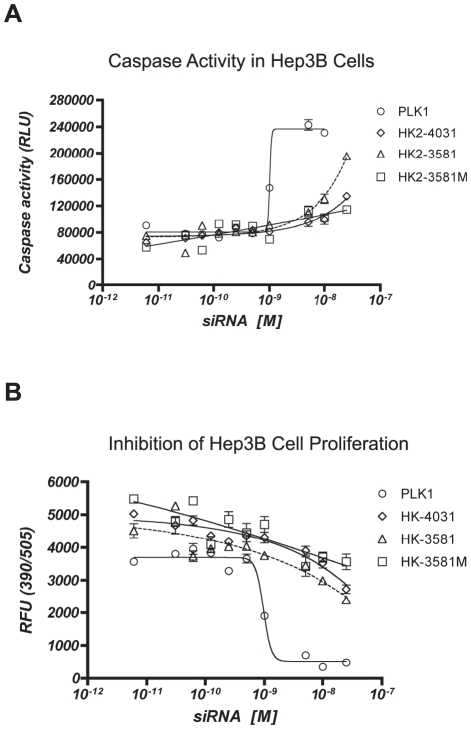
Caspase activity and inhibition of cell proliferation. A) Caspase assays were performed after treating Hep3B cells with 6 pM, 31 pM, 63 pM, 125 pM, 250 pM, 500 pM, 1 nM, 5 nM, 10 nM, and 25 nM of each siRNA. B) Cell proliferation assay were performed for Hep3B cells after treatment with the same concentrations of each siRNA.

Treatment of Hep3B cells with HK2-3581, led to moderate induction of caspase activity at 25 nM (Figure 5A). In contrast, the chemically modified HK2-3581M and HK2-4031 did not induce caspase activity. The same HK2-targeting siRNA did not inhibit cell proliferation relative to a PLK1 siRNA positive control (Figure 5B).

As HK2-4031 did not induce caspase activation, we conclude that caspase activation is independent of HK2 silencing and is most likely due to HK2-3581 off-targets. Indeed, the reduced number of off-targets observed for the lower doses of HK2-3581 (Table 2) was consistent with caspase activation being observed only at the higher dose.

Although the precise mechanism for caspase activation was not determined, we observed two off-targets, BNIP3L and BIRC3, which were significantly down-regulated at higher concentrations (Figures S2-4) and have been previously implicated in apoptosis. BIRC3 (cIAP2) binds to caspase-7 and caspase-3 and protects cells from apoptosis when it is over-expressed [29]. However, BIRC3 is probably not a direct miRNA-like off-target, as its 3′UTR does not have a region that is complementary to the seed of HK2-3581. BNIP3L (BNIP3a, Nix) has been implicated in both positive and negative regulation of apoptosis [30], [31]. As BNIP3L has a 3′UTR that is complementary to the seed of HK2-3581, it is possible that it is a direct miRNA-like off-target.

In summary, the above experiments demonstrate that siRNA off-targets have the potential to cause phenotypic effects at higher doses. The lack of caspase activity observed at the lower doses emphasizes the importance of using the lowest effective dose.

## Discussion

It is now well established that siRNA off-targets exist for many siRNA and that most siRNA molecules are probably not as specific as once thought [32], [33]. Although there are a variety of ways in which off-targets might be induced, miRNA-like binding in the 3′UTR has been proposed as one of the major causes of siRNA off-targets [15], [17]. Consistent with these findings, chemical modification of nucleotides in the seed region of a siRNA can significantly reduce off-targets that are complementary to the seed in the 3′UTR [7], [15], [16], [17], [18], [19]. However, it is also clear that siRNA off-targets can induce detectable phenotypic effects that may or may not be a direct result of 3′UTR binding [20], [34], [35].

This study revealed four key findings that we expect to be applicable to a particular subset of siRNA molecules that potently silence their intended target:

1. Reducing the concentration of a siRNA to a low effective dose where the intended target is still potently silenced can lead to a significant reduction in the number of off-targets that undergo significant changes in expression. This finding was observed for 3 different siRNA duplexes that potently silenced either STAT3 or HK2 (Figures 2-3 and S13). The reduction in off-targets not only applied to transcripts that possessed complementary 3′UTRs but also included other off-targets that did not appear to be the result of a direct miRNA-like interaction. Such off-targets included genes that were annotated as immune response genes, which in the case of STAT3-1676 appeared to be independent of STAT3 silencing.
2. Although we confirmed that the 2′-O-methyl ribosyl modification at position 2 reduced the number of off-targets for one of our two siRNA, several off-targets still existed when cells were treated with a relatively high dose of modified siRNA. Here, the down-regulated off-targets included both transcripts that were complementary to the seed region of the siRNA and transcripts that lacked seed complementarity. Similar to unmodified siRNA, these off-targets were significantly reduced at lower effective doses.
3. Increasing the dose from 10 nM to 25 nM not only caused an increase in the number of down-regulated off-targets that possess 3′UTR complementarity to the seed region, but led to an even more dramatic increase in the number of off-targets that lack 3′UTR complementarity. For example, in HK2-3581 the percentage of 2-fold down-regulated off-targets that possessed seed complementarity in the 3′UTR was 57% at 10 nM and 39% at 25 nM due to a greater increase in the number of off-targets that were not complementary to the seed region at 25 nM (Table 2). A similar trend was observed for STAT3-1676 and STAT3-1676M, and along these lines, k-mer enrichment analysis was usually most striking at 10 nM.
4. A potential phenotypic effect was observed when cells were treated with a relatively high dose (25 nM) of HK2-3581. The moderate induction of apoptosis at the higher dose was also consistent with the increasing number of off-targets observed at increasing doses. Furthermore, the number of off-targets observed for HK2-3581 was much larger than the number of off-targets for other siRNA that targeted HK2. As discussed below, most siRNA screens are performed at concentrations that exceed 25 nM.

While the above findings should not be generalized for all siRNA and their targets, we and others [36] propose that off-targets are best offset using a modified siRNA at the lowest possible dose. Obviously, a siRNA must have a high affinity for its intended target if it is to be effective at a low dose. In contrast, its seed sequence should not bind 3′UTR sequences with high affinity or at least not to 3′UTRs that are amendable to miRNA-like regulation. Determining which seed sequences meet this criteria may not be straightforward but could help design more specific siRNA. While it seems reasonable to avoid seed sequences that are found in endogenous miRNA, it is also worth noting that the presence of a miRNA binding site may be dependent on 3′UTR length and the state of the cells [36].

The concentration of siRNA for most genome-wide and biological screens typically ranges from 25 nM to 100 nM, which is considerably higher than the doses used in this study. Although these high doses improve the sensitivity of a screen, they are likely to increase the number of off-targets. This underscores the need to validate genes detected in genome-wide screens by using additional siRNA molecules that target the same gene.

Our discovery that HK2-3581 induced caspase activity independent of HK2 silencing was serendipitous. Predicting whether observed off-targets induce a significant phenotypic effect is not a trivial problem. Here, the majority of the off-targets were not complementary to the seed region of HK2-3581, and this expression signature might indicate an unanticipated phenotypic effect for a given siRNA. Under this premise, we would expect many of the off-targets to be involved in a common biological process. Indeed, this was the case for HK2-3581, where the up-regulated off-targets were enriched in genes involved in the regulation of cell cycle and were presumably related to the induction of caspase activity. Nevertheless, the prediction of phenotypic effects from a set of observed off-targets remains a difficult problem that warrants further investigation. We eagerly await the development of such methodologies.

In conclusion, siRNA off-targets alter the expression of many genes and have the potential to cause undesirable phenotypic effects. This work demonstrates that is possible reduce and sometimes eliminate off-target effects for both unmodified siRNA and 2′-O-methyl ribosyl modified siRNA, when cells are treated with a relatively low dose of siRNA that is still sufficient to effectively silence the intended target.

## Materials and Methods

### siRNA

The non-targeting control siRNA (siGenome2) and PLK1 siRNA were purchased from Dharmacon/Thermo Scientific Inc. The AllStars negative control was purchased from QIAGEN. The siRNAs that targeted hexokinase II (HK2) and STAT3 were designed to have minimal complementarity with other transcripts encoded by the human genome and were synthesized by Integrated DNA Technologies. The siRNA sequences are described in Table 1.

### Cell culture and transfections

MCF-7 breast cancer cells (American Type Culture Collection) were grown in Gibco RPMI-1640 (Life Technologies) supplemented with 10% fetal calf serum and penicillin/streptomycin. Hep3B cells (American Type Culture Collection) were grown in EMEM (ATCC) supplemented with 10% fetal calf serum. Cells were maintained in monolayer cultures at 37°C in an incubator with 5% CO_2_. One day prior to transfection, cells were seeded at 1.6×10^5^ cells per well in 6 well plates (Real-time PCR and microarrays) and 4×10^3^ cells per well in 96 well plates (Caspase and proliferation assays). Cells were transfected at 30–60% confluence, using Lipofectamine™ RNAiMAX (Life Technologies) according to the manufacturer's instructions and the indicated doses of each siRNA.

Unless otherwise stated, cells were transfected with 0, 1 nM, 10 nM, and 25 nM of siRNA. Given that transfection efficiencies can vary according to the ratio of nucleotide to transfection reagent, each dose was supplemented with non-targeting (negative) control siRNA, such that the total RNA concentration was equal across experiments. For example, in the microarray experiment where the concentrations of STAT3-1676 were 0, 1 nM, 10 nM, and 25 nM, we added 25 nM, 24 nM, 15 nM, and 0 nM of siGenome2 respectively. RNA was purified 48 hours post-transfection using the RNeasy mini kit from Qiagen.

### Real-time PCR

Three biological replicates were used to assess silencing of the intended targets by real-time PCR. Primers for STAT3 and HK2 were obtained from Applied Biosystems (TaqMan® Gene Expression Assays). Real-time PCR was performed in a total volume of 25 µL, using the Universal PCR Master Mix as described in the manufacturers protocol. Relative mRNA levels were calculated as 2^−ΔΔCt^ values.

### Microarray experiments and computational analysis

Off-targets were assessed using the Affymetrix gene expression platform and all data is MIAME compliant and available at GEO (GSE28786). Biological triplicates were used unless specifically stated. RNA was hybridized to Affymetrix human genome U133 plus 2.0 arrays. The arrays were scanned using an Affymetrix GeneChip Scanner 3000. Probesets were mapped to Entrez Gene IDs using custom CDF files [37]. Affymetrix CEL files were normalized in Bioconductor [38] using the RMA method [39]. Differentially regulated genes were identified using a moderated t-test [40]. False discovery rate adjusted P-values were calculated using the method of Benjamini and Hochberg [41]. Genes were mapped to GO terms using the AnnotationDbi package and GO enrichment analysis was performed using the GOstats package [42]. The longest 3′UTR sequence for each human gene was retrieved using the ENSEMBL API [43] and k-mer enrichment analysis was performed using an implementation of the Sylamer method [26].

### Cell proliferation assays

On assay day, 10 ml of CellTiter-Fluor reagent mix (Promega, cat#G6081) was added to each well on cell plates. After briefly shaking at gentle speed, plates were incubated at 37°C for 30 min before fluorescence signals were measured using a SpectraMax M5 Microplate Reader(Molecular Devices, CA) at 390ex/505em with autocutoff  = 495. Fluorescence data were recorded for cell proliferation analysis. Linearity of the CellTiter-Fluor assay with cell number was tested routinely in each experiment and used for getting a conversion formula of fluorescence signals for calculating the relative cell numbers.

### Caspase assays

Within 1 hour of completing the CellTiter-Fluor assay for cell proliferation, 96 ml of Caspase3/7 reagent mix (Promega, cat#G8091) was added to each well of the cell plates. Plates were shaken gently at room temperature for 1 hr before reading of luminescence signals (caspase activity) with an Envision plate reader.

## Supporting Information

Figure S1 **Off-targets for STAT3-1676.** A) Cells were transfected with 25 nM of siRNA and a relatively large number of off-targets were detected. B) Cells were transfected with 10 nM of siRNA and only one of the off-targets was down-regulated more than the intended target. C) Cells were transfected with 1 nM of siRNA and none of the off-targets were down-regulated more than the intended target. Off-targets are defined as transcripts with a 2-fold change in mRNA levels for one or more concentrations. The entire set of off-targets are plotted at each concentration. The volcano plots consist of Log_2_ fold-change values between control siRNA and STAT3-1676 on the x-axis and log10 P-values from the moderated T-test on the y-axis. Off-targets that possess 3′UTRs that are complementary to the seed region (position 2–6 of the siRNA) are indicated with a blue diamond. Immune response genes are indicated with a red circle. Other off-targets and the intended target are indicated according to the legend.
(EPS)

Figure S2 **Off-targets for HK2-3581.** A) Cells were transfected with 25 nM of siRNA and a relatively large number of off-targets were detected. B) Cells were transfected with 10 nM of siRNA and only one off-target was down-regulated more than the intended target. C) Cells were transfected with 1 nM of siRNA and none of the off-targets were down-regulated more than the intended target. Off-targets are plotted as described in Figure S1.
(EPS)

Figure S3 **Off-targets for modified STAT3-1676M.** A) Cells were transfected with 25 nM of siRNA and a relatively large number of off-targets were detected. B) Cells were transfected with 10 nM of siRNA and only one off-target was down-regulated more than the intended target. C) Cells were transfected with 1 nM of siRNA and none of the off-targets were down-regulated more than the intended target. Off-targets are plotted as described in Figure S1.
(EPS)

Figure S4 **Off-targets for modified HK2-3581M.** A) Cells were transfected with 25 nM of siRNA and a large number of off-targets were detected. B) Cells were transfected with 10 nM of siRNA and none of the off-targets were down-regulated more than the intended target. C) Cells were transfected with 1 nM of siRNA and only one of the off-targets was down-regulated more than the intended target. Off-targets are plotted as described in Figure S1.
(EPS)

Figure S5 **3′UTR hexamer enrichment analysis for STAT3-1676.** A) 25 nM. B) 10 nM. C) 1 nM. Transcripts from each microarray were rank-ordered by log_2_ fold-change and P-values were computed at different levels of fold-change (increments of 100). The hyper-geometric test was used to assess whether a particular hexamer was over or under-represented in 3′UTRs at each level of fold-change.
(TIF)

Figure S6 **3′UTR heptamer enrichment analysis for STAT3-1676.** A) 25 nM. B) 10 nM. C) 1 nM. Transcripts from each microarray were rank-ordered by log_2_ fold-change and P-values were computed at different levels of fold-change (increments of 100). The hyper-geometric test was used to assess whether a particular heptamer was over or under-represented in 3′UTRs at each level of fold-change.
(TIF)

Figure S7 **3′UTR hexamer enrichment analysis for STAT3-1676M.** A) 25 nM. B) 10 nM. C) 1 nM. Transcripts from each microarray were rank-ordered by log_2_ fold-change and P-values were computed at different levels of fold-change (increments of 100). The hyper-geometric test was used to assess whether a particular hexamer was over or under-represented in 3′UTRs at each level of fold-change.
(TIF)

Figure S8 **3′UTR heptamer enrichment analysis for STAT3-1676M.** A) 25 nM. B) 10 nM. C) 1 nM. Transcripts from each microarray were rank-ordered by log_2_ fold-change and P-values were computed at different levels of fold-change (increments of 100). The hyper-geometric test was used to assess whether a particular heptamer was over or under-represented in 3′UTRs at each level of fold-change.
(TIF)

Figure S9 **3′UTR hexamer enrichment analysis for HK2-3581.** A) 25 nM. B) 10 nM. C) 1 nM. Transcripts from each microarray were rank-ordered by log_2_ fold-change and P-values were computed at different levels of fold-change (increments of 100). The hyper-geometric test was used to assess whether a particular hexamer was over or under-represented in 3′UTRs at each level of fold-change.
(TIF)

Figure S10 **3′UTR heptamer enrichment analysis for HK2-3581.** A) 25 nM. B) 10 nM. C) 1 nM. Transcripts from each microarray were rank-ordered by log_2_ fold-change and P-values were computed at different levels of fold-change (increments of 100). The hyper-geometric test was used to assess whether a particular heptamer was over or under-represented in 3′UTRs at each level of fold-change.
(TIF)

Figure S11 **3′UTR hexamer enrichment analysis for HK2-3581M.** A) 25 nM. B) 10 nM. C) 1 nM. Transcripts from each microarray were rank-ordered by log_2_ fold-change and P-values were computed at different levels of fold-change (increments of 100). The hyper-geometric test was used to assess whether a particular hexamer was over or under-represented in 3′UTRs at each level of fold-change.
(TIF)

Figure S12 **3′UTR heptamer enrichment analysis for HK2-3581M.** A) 25 nM. B) 10 nM. C) 1 nM. Transcripts from each microarray were rank-ordered by log_2_ fold-change and P-values were computed at different levels of fold-change (increments of 100). The hyper-geometric test was used to assess whether a particular heptamer was over or under-represented in 3′UTRs at each level of fold-change.
(TIF)

Figure S13 **Off-targets for HK2-4031.** A) Cells were transfected with 10 nM of HK2-4031 and 0 nM of AllStars negative control. B) Cells were transfected with 1 nM of HK2-4031 and 9 nM of AllStars negative control. Off-targets are defined as transcripts with a 2-fold change in mRNA levels for one or more concentrations. The entire set of off-targets are plotted at each concentration. The volcano plots consist of Log_2_ fold-change values between control siRNA and STAT3-1676 on the x-axis and P-values from the moderated T-test on the y-axis. Off-targets that possess 3′UTRs that are complementary to the seed region (position 2–6 of the siRNA) are indicated with a blue diamond. Other off-targets and the intended target are indicated according to the legend.
(EPS)

Table S1 **STAT3-1676 off-targets that are involved in immune response.**
(DOC)

Table S2 **Enrichment of STAT3-1676 off-targets that are involved in immune response.**
(DOC)

Table S3 **HK2-3581 off-targets that are involved in immune response.**
(DOC)

Table S4 **Enrichment of HK2-3581 off-targets that are involved in cell cycle.**
(DOC)

Table S5 **HK2-3581 off-targets that are involved in cell cycle.**
(DOC)

Table S6 **Enrichment of HK2-3581M off-targets that are involved in cell cycle.**
(DOC)

Table S7 **HK2-3581M off-targets that are involved in cell cycle.**
(DOC)
