# Supplementary figures and images for: siRNA Off-Target Effects Can Be Reduced at Concentrations That Match Their Individual Potency
Source: PLoS One. 2011 Jul 5;6(7):e21503. doi: 10.1371/journal.pone.0021503 (PMC3130022; doi:10.1371/journal.pone.0021503)

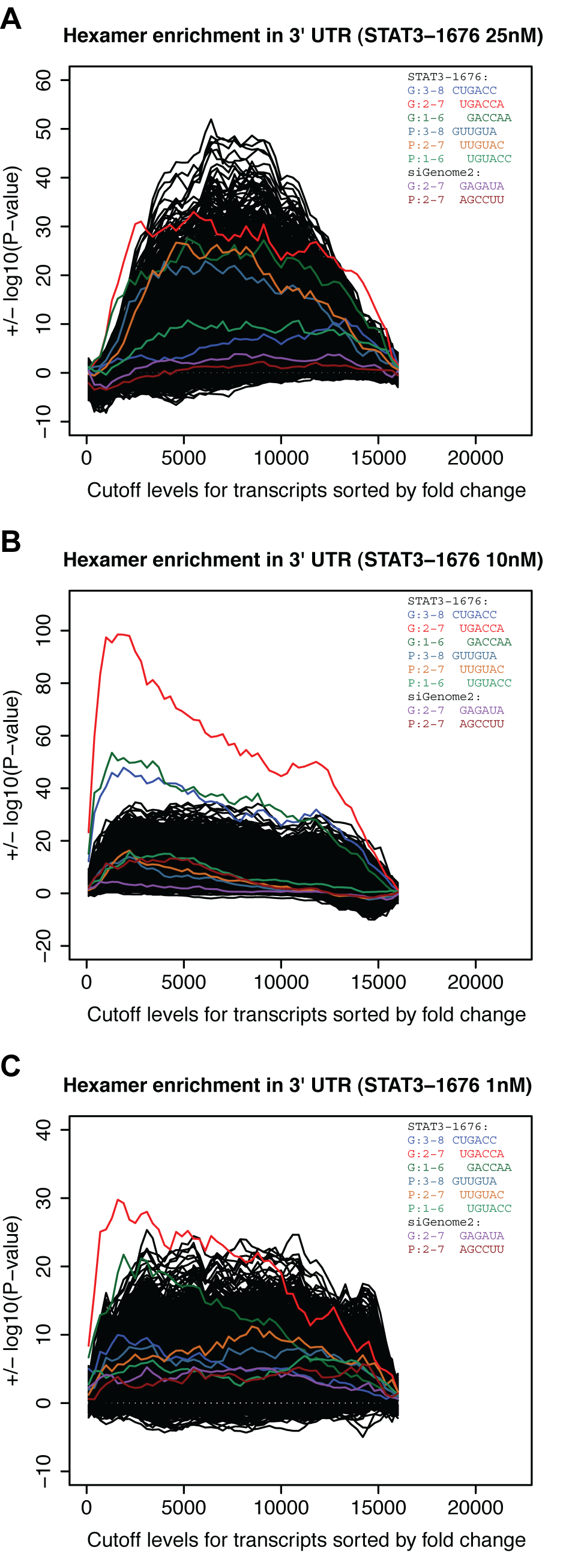

Supplement: Figure S5 — 3′UTR hexamer enrichment analysis for STAT3-1676. A) 25 nM. B) 10 nM. C) 1 nM. Transcripts from each microarray were rank-ordered by log2 fold-change and P-values were computed at different levels of fold-change (increments of 100). The hyper-geometric test was used to assess whether a particular hexamer was over or under-represented in 3′UTRs at each level of fold-change. (TIF) [file pone.0021503.s005.tif]

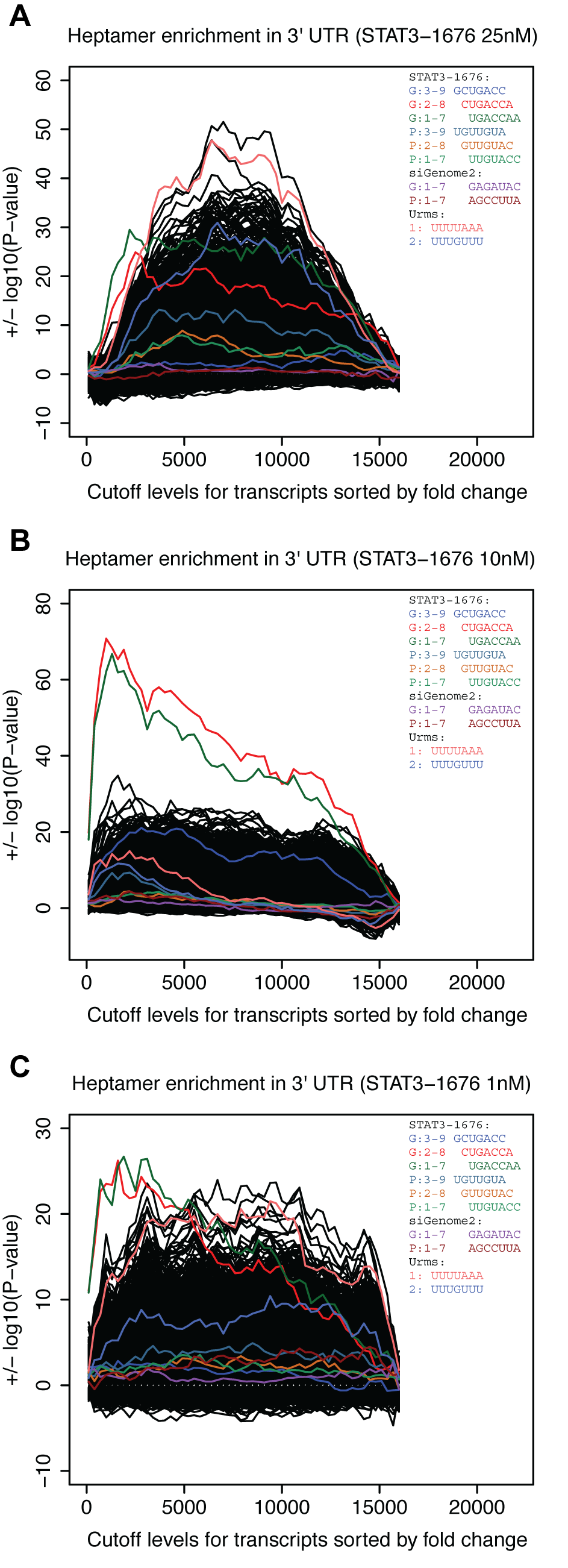

Supplement: Figure S6 — 3′UTR heptamer enrichment analysis for STAT3-1676. A) 25 nM. B) 10 nM. C) 1 nM. Transcripts from each microarray were rank-ordered by log2 fold-change and P-values were computed at different levels of fold-change (increments of 100). The hyper-geometric test was used to assess whether a particular heptamer was over or under-represented in 3′UTRs at each level of fold-change. (TIF) [file pone.0021503.s006.tif]

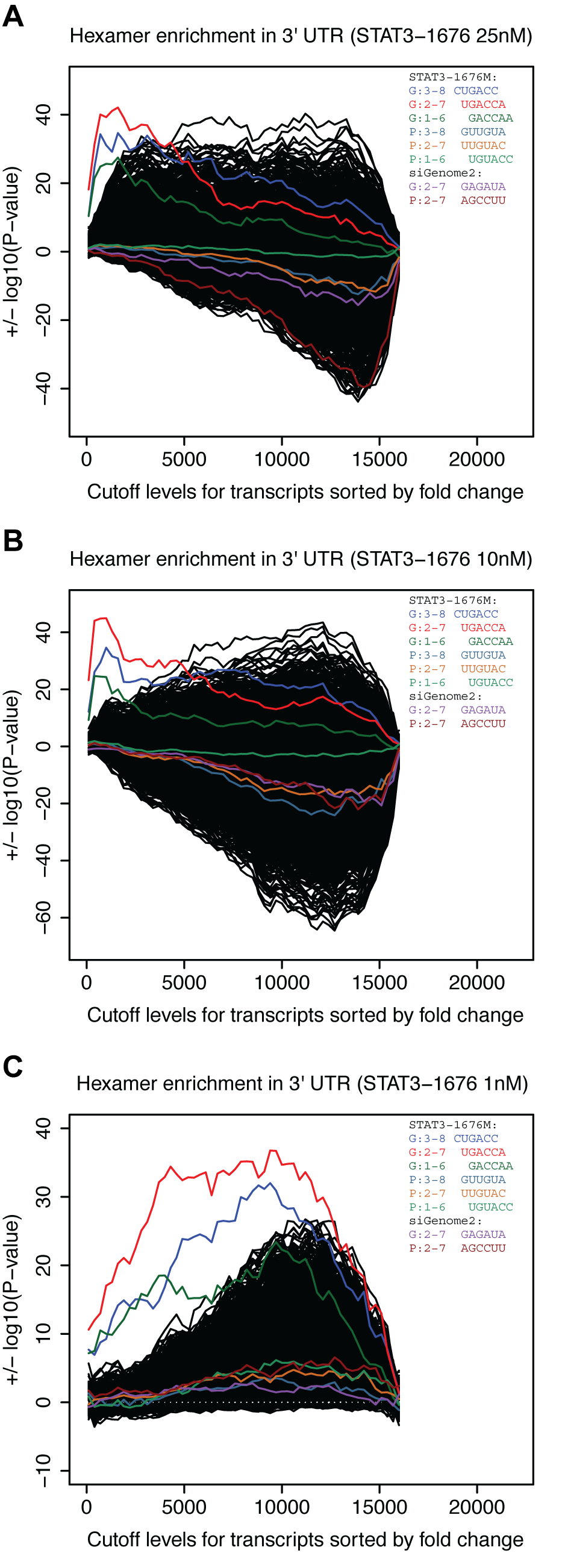

Supplement: Figure S7 — 3′UTR hexamer enrichment analysis for STAT3-1676M. A) 25 nM. B) 10 nM. C) 1 nM. Transcripts from each microarray were rank-ordered by log2 fold-change and P-values were computed at different levels of fold-change (increments of 100). The hyper-geometric test was used to assess whether a particular hexamer was over or under-represented in 3′UTRs at each level of fold-change. (TIF) [file pone.0021503.s007.tif]

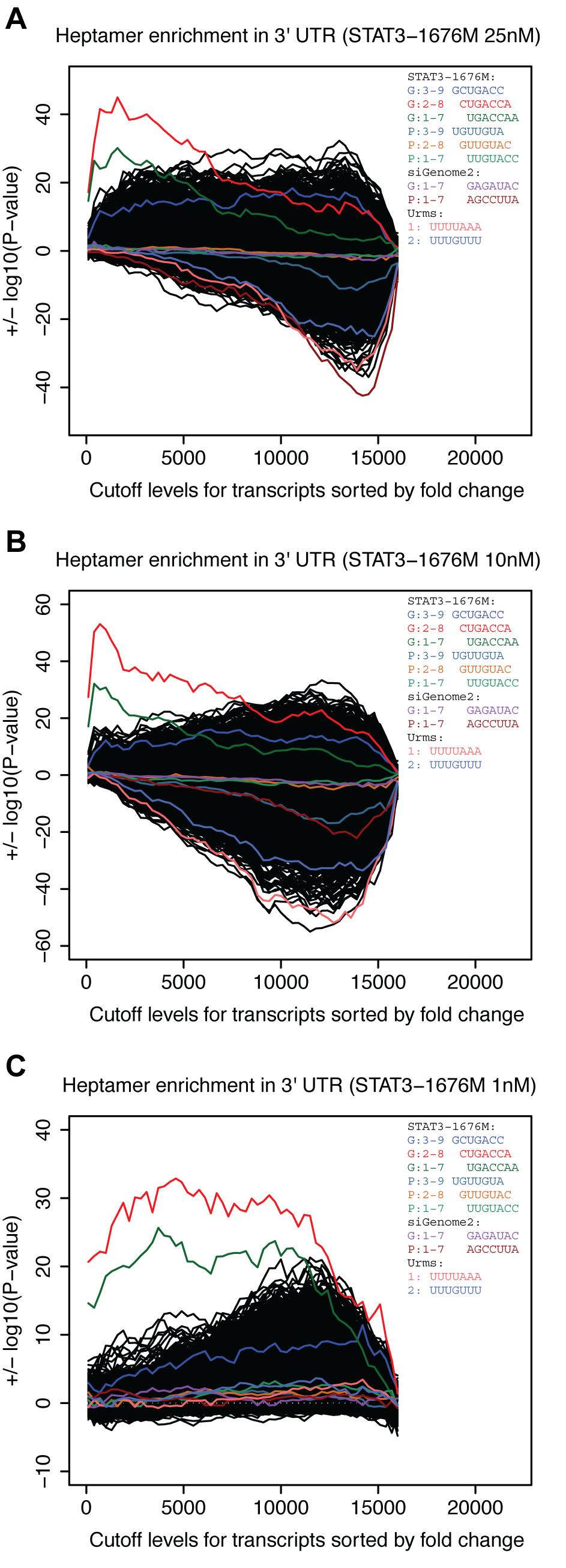

Supplement: Figure S8 — 3′UTR heptamer enrichment analysis for STAT3-1676M. A) 25 nM. B) 10 nM. C) 1 nM. Transcripts from each microarray were rank-ordered by log2 fold-change and P-values were computed at different levels of fold-change (increments of 100). The hyper-geometric test was used to assess whether a particular heptamer was over or under-represented in 3′UTRs at each level of fold-change. (TIF) [file pone.0021503.s008.tif]

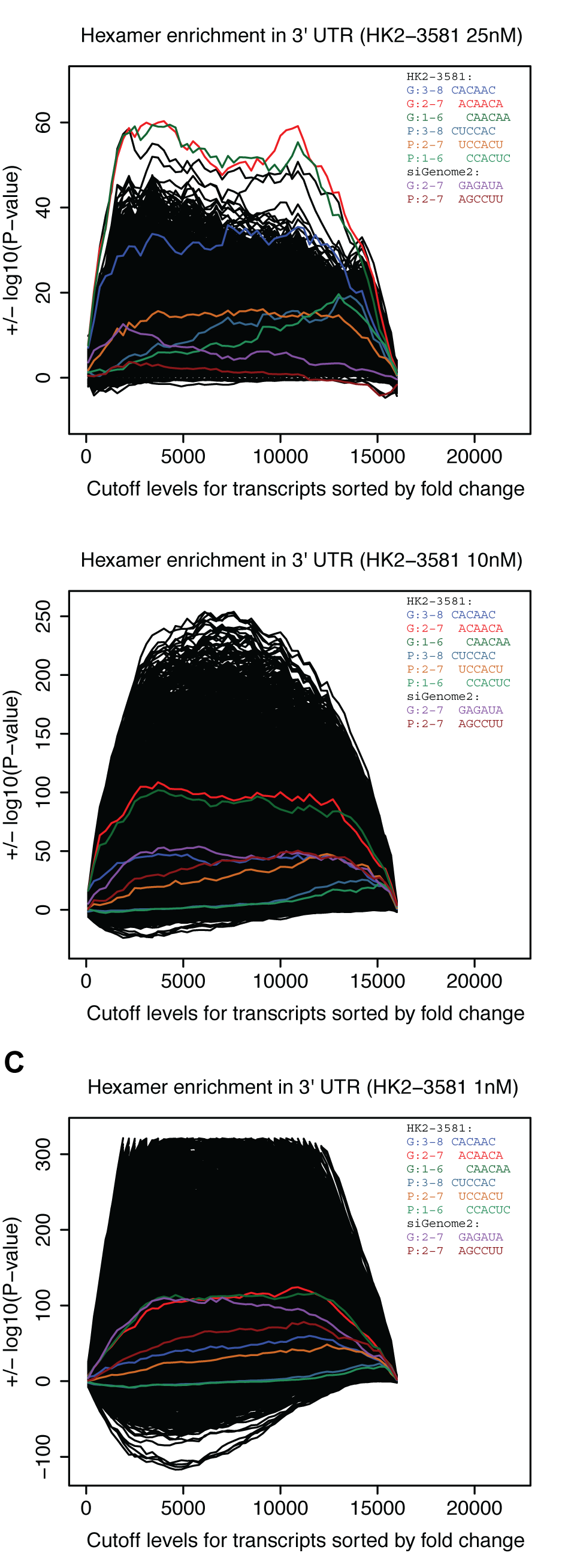

Supplement: Figure S9 — 3′UTR hexamer enrichment analysis for HK2-3581. A) 25 nM. B) 10 nM. C) 1 nM. Transcripts from each microarray were rank-ordered by log2 fold-change and P-values were computed at different levels of fold-change (increments of 100). The hyper-geometric test was used to assess whether a particular hexamer was over or under-represented in 3′UTRs at each level of fold-change. (TIF) [file pone.0021503.s009.tif]

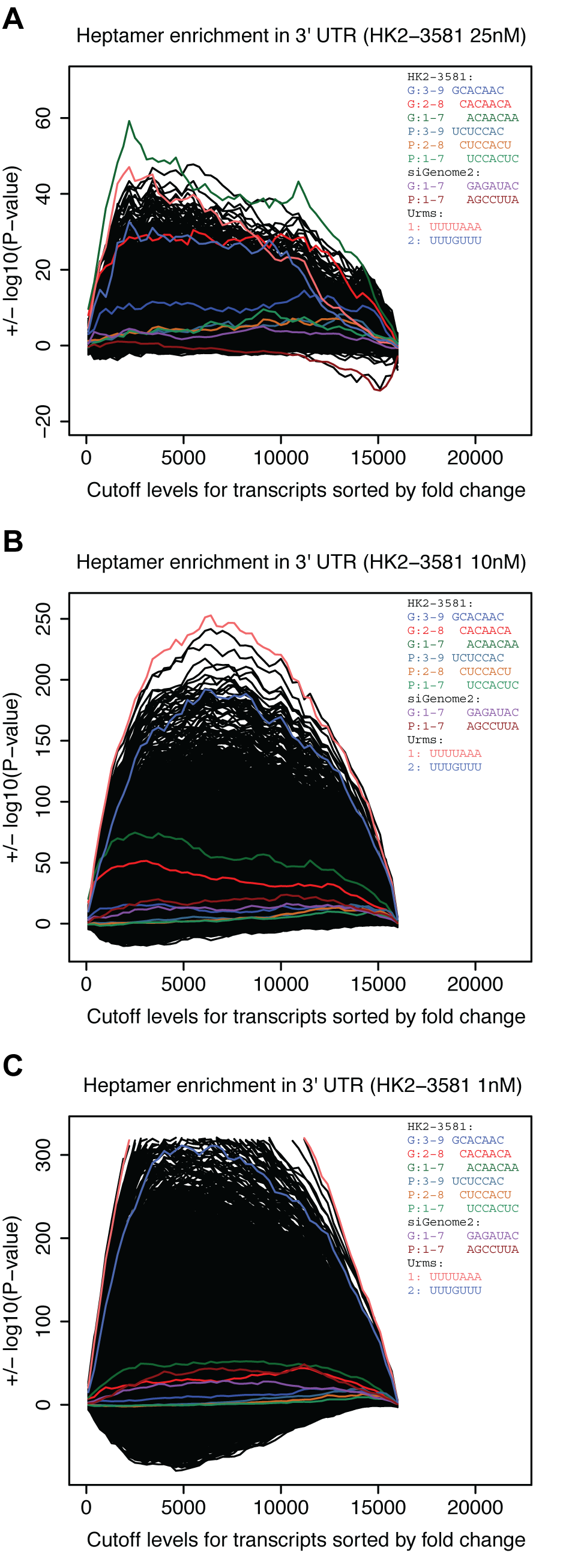

Supplement: Figure S10 — 3′UTR heptamer enrichment analysis for HK2-3581. A) 25 nM. B) 10 nM. C) 1 nM. Transcripts from each microarray were rank-ordered by log2 fold-change and P-values were computed at different levels of fold-change (increments of 100). The hyper-geometric test was used to assess whether a particular heptamer was over or under-represented in 3′UTRs at each level of fold-change. (TIF) [file pone.0021503.s010.tif]

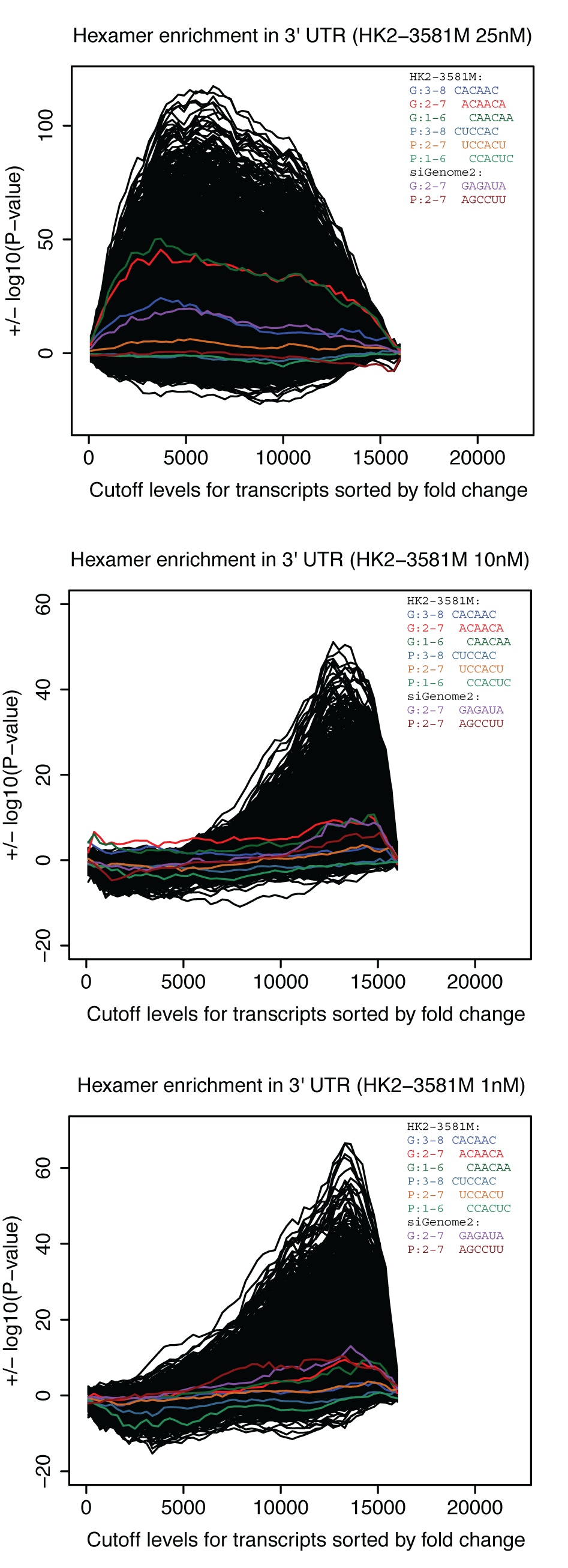

Supplement: Figure S11 — 3′UTR hexamer enrichment analysis for HK2-3581M. A) 25 nM. B) 10 nM. C) 1 nM. Transcripts from each microarray were rank-ordered by log2 fold-change and P-values were computed at different levels of fold-change (increments of 100). The hyper-geometric test was used to assess whether a particular hexamer was over or under-represented in 3′UTRs at each level of fold-change. (TIF) [file pone.0021503.s011.tif]

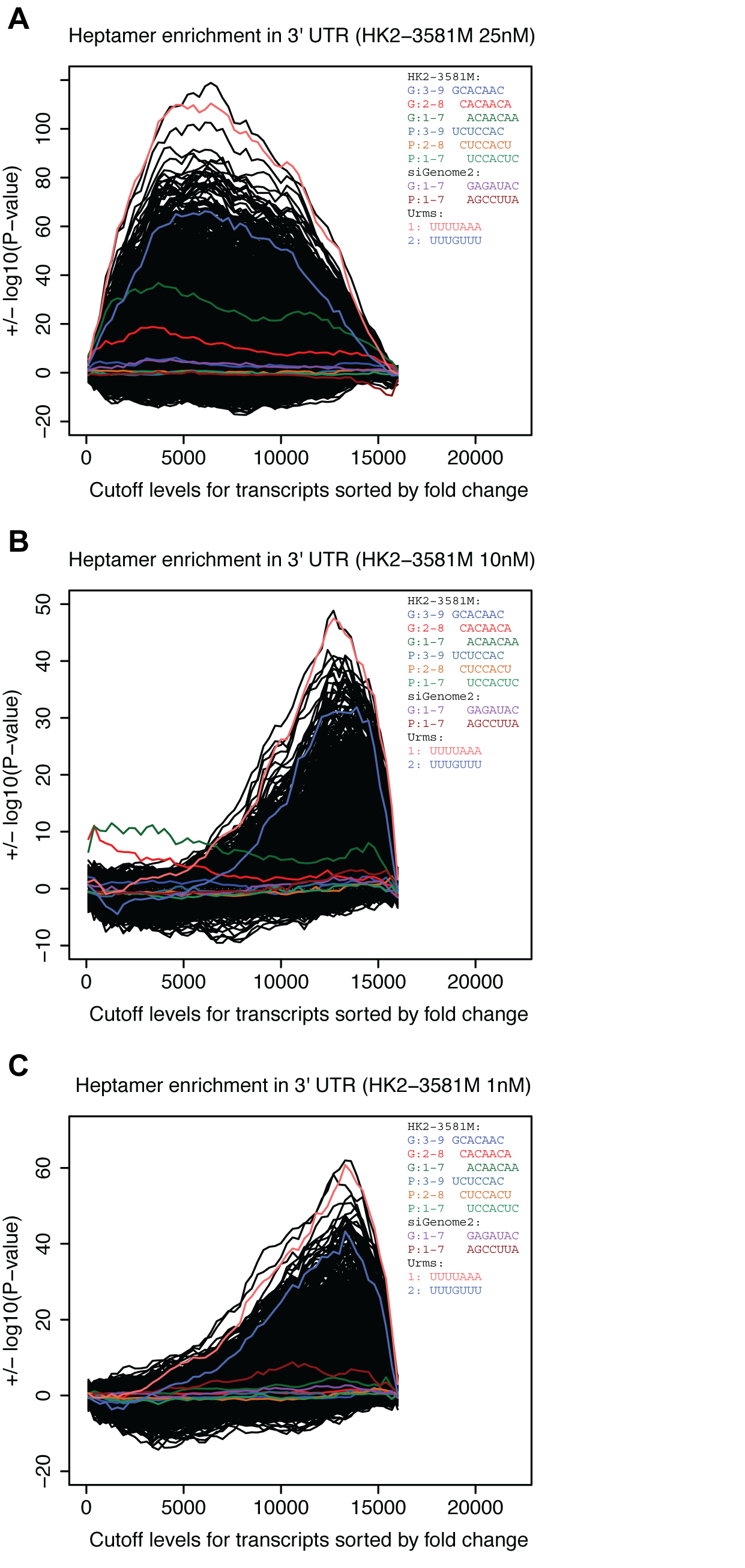

Supplement: Figure S12 — 3′UTR heptamer enrichment analysis for HK2-3581M. A) 25 nM. B) 10 nM. C) 1 nM. Transcripts from each microarray were rank-ordered by log2 fold-change and P-values were computed at different levels of fold-change (increments of 100). The hyper-geometric test was used to assess whether a particular heptamer was over or under-represented in 3′UTRs at each level of fold-change. (TIF) [file pone.0021503.s012.tif]
