# Supplementary material for: siRNA Off-Target Effects Can Be Reduced at Concentrations That Match Their Individual Potency
Source: PLoS One. 2011 Jul 5;6(7):e21503. doi: 10.1371/journal.pone.0021503 (PMC3130022; doi:10.1371/journal.pone.0021503)
Supplement: Table S1 — STAT3-1676 off-targets that are involved in immune response. (DOC) [file pone.0021503.s014.doc]

**Table S1.** STAT3-1676 off-targets that are involved in immune response.

| geneSymbol | entrezGeneId | 25nMLog_2_Fc | 10nMLog_2_Fc | 1nMLog_2_Fc |
| --- | --- | --- | --- | --- |
| OAS2 | 4939 | 1.524595734 | 1.06209968 | 0.129463792 |
| IFI44L | 10964 | 1.444183569 | 1.071088477 | 0.399673082 |
| OAS1 | 4938 | 1.254532995 | 1.38786655 | 0.522335967 |
| PSMB8 | 5696 | 1.239714123 | 1.053349908 | 0.466652054 |
| GBP3 | 2635 | 1.076726563 | 1.616876526 | 0.543179138 |
| BST2 | 684 | 1.056390779 | 0.782622152 | 0.348673547 |
| PSMB9 | 5698 | 1.049637083 | 1.141869956 | 0.353131774 |
| TAP1 | 6890 | 1.019296121 | 1.335862796 | 0.354646 |
| OASL | 8638 | 1.008760971 | 0.988026466 | 0.345528529 |
| HMOX1 | 3162 | 0.694982779 | 1.772585568 | 0.49938156 |
| PROCR | 10544 | 0.583754807 | 1.250626847 | 0.56306435 |
| TICAM1 | 148022 | 0.443645669 | 1.021808682 | 0.407184638 |
| RELB | 5971 | 0.329396358 | 1.705646645 | 0.347989496 |
| GBP2 | 2634 | 0.218091406 | 1.099663225 | 0.305553748 |
| ICAM1 | 3383 | 0.131392765 | 1.752285542 | 0.471812126 |
| TNF | 7124 | 0.032518996 | 2.155787758 | 0.526014866 |
| GBP1 | 2633 | 0.013399849 | 1.942517912 | 0.233470152 |
| CXCL2 | 2920 | -0.013061782 | 1.387781992 | 0.11009331 |
| IL8 | 3576 | -0.229382971 | 1.723192249 | 0.019283418 |
| CCL20 | 6364 | -0.238996778 | 3.169210848 | 0.113209747 |
| BCL3 | 602 | -0.306344498 | 1.009064179 | 0.312051674 |
| CFB | 629 | -0.354034362 | 3.004599228 | 0.688424239 |
| NFKBIA | 4792 | -0.973462158 | 1.433026583 | 0.279411116 |
| PTMS | 5763 | -1.054826459 | -0.372193527 | -0.246759238 |

All up-regulated and down-regulated off-targets annotated as immune response genes (GO:0006955) are described along with log_2_ fold-change values at each concentration. The majority of these genes are down-regulated.
