# Supplementary material for: siRNA Off-Target Effects Can Be Reduced at Concentrations That Match Their Individual Potency
Source: PLoS One. 2011 Jul 5;6(7):e21503. doi: 10.1371/journal.pone.0021503 (PMC3130022; doi:10.1371/journal.pone.0021503)
Supplement: Table S2 — Enrichment of STAT3-1676 off-targets that are involved in immune response. (DOC) [file pone.0021503.s015.doc]

**Table S2.** Enrichment of STAT3-1676 off-targets that are involved in immune response.

| GOBPID | Pvalue | OddsRatio | ExpCount | Count | Size | Term |
| --- | --- | --- | --- | --- | --- | --- |
| GO:0006955 | 1.61E-13 | 9.56095679 | 3.494917068 | 23 | 644 | immune response |
| GO:0002376 | 6.38E-13 | 7.822271881 | 4.998165558 | 26 | 921 | immune system process |
| GO:0050896 | 5.83E-09 | 4.197422882 | 14.09363296 | 36 | 2597 | response to stimulus |
| GO:0007249 | 9.96E-07 | 12.11240447 | 0.77604525 | 8 | 143 | I-kappaB kinase/NF-kappaB cascade |
| GO:0043122 | 1.55E-05 | 12.82282878 | 0.537262096 | 6 | 99 | regulation of I-kappaB kinase/NF-kappaB cascade |
| GO:0009611 | 3.64E-05 | 5.09439528 | 2.512650004 | 11 | 463 | response to wounding |
| GO:0006952 | 5.24E-05 | 4.52251816 | 3.104180998 | 12 | 572 | defense response |
| GO:0002286 | 5.34E-05 | 52.14304813 | 0.075976458 | 3 | 14 | T cell activation during immune response |
| GO:0042345 | 5.34E-05 | 52.14304813 | 0.075976458 | 3 | 14 | regulation of NF-kappaB import into nucleus |
| GO:0042348 | 5.34E-05 | 52.14304813 | 0.075976458 | 3 | 14 | NF-kappaB import into nucleus |
| GO:0042108 | 9.18E-05 | 19.36119403 | 0.238783154 | 4 | 44 | positive regulation of cytokine biosynthetic process |
| GO:0002285 | 0.000117885 | 38.22647059 | 0.097684017 | 3 | 18 | lymphocyte activation during immune response |
| GO:0043123 | 0.000132151 | 11.38653982 | 0.493846977 | 5 | 91 | positive regulation of I-kappaB kinase/NF-kappaB cascade |
| GO:0006950 | 0.000147984 | 3.020929213 | 7.722464267 | 19 | 1423 | response to stress |
| GO:0002263 | 0.000163415 | 33.72404844 | 0.108537797 | 3 | 20 | cell activation during immune response |
| GO:0002366 | 0.000163415 | 33.72404844 | 0.108537797 | 3 | 20 | leukocyte activation during immune response |
| GO:0030155 | 0.000178587 | 10.63899868 | 0.526408316 | 5 | 97 | regulation of cell adhesion |
| GO:0001775 | 0.000188545 | 5.630219568 | 1.600932508 | 8 | 295 | cell activation |
| GO:0051707 | 0.00018966 | 6.541033879 | 1.199342658 | 7 | 221 | response to other organism |
| GO:0019882 | 0.000190781 | 15.79409077 | 0.287625162 | 4 | 53 | antigen processing and presentation |
| GO:0006915 | 0.000203534 | 3.487948844 | 4.721394176 | 14 | 870 | apoptosis |
| GO:0012501 | 0.000229181 | 3.444836109 | 4.775663074 | 14 | 880 | programmed cell death |
| GO:0050900 | 0.000236383 | 14.87944891 | 0.303905832 | 4 | 56 | leukocyte migration |
| GO:0009607 | 0.000252772 | 5.380740741 | 1.671482076 | 8 | 308 | response to biotic stimulus |
| GO:0002521 | 0.000267327 | 7.461828737 | 0.895436826 | 6 | 165 | leukocyte differentiation |
| GO:0006954 | 0.000275806 | 5.308270677 | 1.693189635 | 8 | 312 | inflammatory response |
| GO:0009615 | 0.000307872 | 9.402680653 | 0.591530994 | 5 | 109 | response to virus |
| GO:0042990 | 0.000364099 | 24.91496164 | 0.141099136 | 3 | 26 | regulation of transcription factor import into nucleus |
| GO:0042991 | 0.000364099 | 24.91496164 | 0.141099136 | 3 | 26 | transcription factor import into nucleus |
| GO:0046632 | 0.000364099 | 24.91496164 | 0.141099136 | 3 | 26 | alpha-beta T cell differentiation |
| GO:0030097 | 0.000421247 | 5.699553571 | 1.367576244 | 7 | 252 | hemopoiesis |
| GO:0042035 | 0.000471647 | 12.27102582 | 0.36360162 | 4 | 67 | regulation of cytokine biosynthetic process |
| GO:0045321 | 0.000507929 | 5.515872036 | 1.410991363 | 7 | 260 | leukocyte activation |
| GO:0009605 | 0.000542322 | 3.457064491 | 3.988764045 | 12 | 735 | response to external stimulus |
| GO:0009651 | 0.000599171 | 75.40289855 | 0.037988229 | 2 | 7 | response to salt stress |
| GO:0045080 | 0.000599171 | 75.40289855 | 0.037988229 | 2 | 7 | positive regulation of chemokine biosynthetic process |
| GO:0008219 | 0.000610121 | 3.100909341 | 5.258656271 | 14 | 969 | cell death |
| GO:0002573 | 0.000620286 | 11.3643547 | 0.39073607 | 4 | 72 | myeloid leukocyte differentiation |
| GO:0016265 | 0.000635647 | 3.086950954 | 5.280363831 | 14 | 973 | death |
| GO:0048534 | 0.000635717 | 5.301984316 | 1.465260261 | 7 | 270 | hemopoietic or lymphoid organ development |
| GO:0000060 | 0.000678581 | 19.7510142 | 0.173660475 | 3 | 32 | protein import into nucleus, translocation |
| GO:0046631 | 0.000678581 | 19.7510142 | 0.173660475 | 3 | 32 | alpha-beta T cell activation |
| GO:0006959 | 0.000688114 | 11.03795309 | 0.401589849 | 4 | 74 | humoral immune response |
| GO:0007243 | 0.000720553 | 4.118582975 | 2.452954215 | 9 | 452 | protein kinase cascade |
| GO:0045123 | 0.00079609 | 62.83091787 | 0.043415119 | 2 | 8 | cellular extravasation |
| GO:0042089 | 0.000799476 | 10.5818851 | 0.417870519 | 4 | 77 | cytokine biosynthetic process |
| GO:0042107 | 0.000839267 | 10.43807987 | 0.423297409 | 4 | 78 | cytokine metabolic process |
| GO:0007267 | 0.000882573 | 3.686453217 | 3.060765879 | 10 | 564 | cell-cell signaling |
| GO:0010876 | 0.000885056 | 17.89522059 | 0.189941145 | 3 | 35 | lipid localization |
| GO:0019915 | 0.000885056 | 17.89522059 | 0.189941145 | 3 | 35 | lipid storage |
| GO:0002520 | 0.000892195 | 4.991655466 | 1.552090499 | 7 | 286 | immune system development |
| GO:0051704 | 0.00091909 | 3.665715043 | 3.077046549 | 10 | 567 | multi-organism process |
| GO:0010627 | 0.00100562 | 5.738312173 | 1.15050065 | 6 | 212 | regulation of protein kinase cascade |
| GO:0042306 | 0.001127566 | 16.35756303 | 0.206221815 | 3 | 38 | regulation of protein import into nucleus |
| GO:0010740 | 0.001200525 | 6.866197183 | 0.797752809 | 5 | 147 | positive regulation of protein kinase cascade |
| GO:0051101 | 0.001262553 | 9.299766229 | 0.472139418 | 4 | 87 | regulation of DNA binding |
| GO:0045073 | 0.001270471 | 47.11594203 | 0.054268899 | 2 | 10 | regulation of chemokine biosynthetic process |
| GO:0002252 | 0.001518785 | 6.495959596 | 0.841167928 | 5 | 155 | immune effector process |
| GO:0002292 | 0.001547354 | 41.87761675 | 0.059695788 | 2 | 11 | T cell differentiation during immune response |
| GO:0002293 | 0.001547354 | 41.87761675 | 0.059695788 | 2 | 11 | alpha-beta T cell differentiation during immune response |
| GO:0002294 | 0.001547354 | 41.87761675 | 0.059695788 | 2 | 11 | CD4-positive, alpha-beta T cell differentiation during immune response |
| GO:0042033 | 0.001547354 | 41.87761675 | 0.059695788 | 2 | 11 | chemokine biosynthetic process |
| GO:0042093 | 0.001547354 | 41.87761675 | 0.059695788 | 2 | 11 | T-helper cell differentiation |
| GO:0045069 | 0.001547354 | 41.87761675 | 0.059695788 | 2 | 11 | regulation of viral genome replication |
| GO:0050755 | 0.001547354 | 41.87761675 | 0.059695788 | 2 | 11 | chemokine metabolic process |
| GO:0033157 | 0.001729511 | 13.95731707 | 0.238783154 | 3 | 44 | regulation of intracellular protein transport |
| GO:0050776 | 0.001795397 | 6.243201243 | 0.873729267 | 5 | 161 | regulation of immune response |
| GO:0042088 | 0.001850318 | 37.68695652 | 0.065122678 | 2 | 12 | T-helper 1 type immune response |
| GO:0002274 | 0.002093029 | 13.0026738 | 0.255063823 | 3 | 47 | myeloid leukocyte activation |
| GO:0002761 | 0.002359826 | 12.43542199 | 0.265917603 | 3 | 49 | regulation of myeloid leukocyte differentiation |
| GO:0009628 | 0.00237436 | 4.810172684 | 1.362149354 | 6 | 251 | response to abiotic stimulus |
| GO:0046822 | 0.002500758 | 12.16989987 | 0.271344493 | 3 | 50 | regulation of nucleocytoplasmic transport |
| GO:0002685 | 0.002533357 | 31.40096618 | 0.075976458 | 2 | 14 | regulation of leukocyte migration |
| GO:0032642 | 0.002533357 | 31.40096618 | 0.075976458 | 2 | 14 | regulation of chemokine production |
| GO:0045682 | 0.002533357 | 31.40096618 | 0.075976458 | 2 | 14 | regulation of epidermis development |
| GO:0040011 | 0.002816295 | 3.636836967 | 2.425819766 | 8 | 447 | locomotion |
| GO:0002718 | 0.002912872 | 28.98327759 | 0.081403348 | 2 | 15 | regulation of cytokine production during immune response |
| GO:0006970 | 0.002912872 | 28.98327759 | 0.081403348 | 2 | 15 | response to osmotic stress |
| GO:0032602 | 0.002912872 | 28.98327759 | 0.081403348 | 2 | 15 | chemokine production |
| GO:0043331 | 0.002912872 | 28.98327759 | 0.081403348 | 2 | 15 | response to dsRNA |
| GO:0048513 | 0.003305511 | 2.409026067 | 7.695329817 | 16 | 1418 | organ development |
| GO:0001836 | 0.003317349 | 26.91097308 | 0.086830238 | 2 | 16 | release of cytochrome c from mitochondria |
| GO:0015800 | 0.003317349 | 26.91097308 | 0.086830238 | 2 | 16 | acidic amino acid transport |
| GO:0015813 | 0.003317349 | 26.91097308 | 0.086830238 | 2 | 16 | L-glutamate transport |
| GO:0043367 | 0.003317349 | 26.91097308 | 0.086830238 | 2 | 16 | CD4-positive, alpha beta T cell differentiation |
| GO:0045429 | 0.003317349 | 26.91097308 | 0.086830238 | 2 | 16 | positive regulation of nitric oxide biosynthetic process |
| GO:0048168 | 0.003317349 | 26.91097308 | 0.086830238 | 2 | 16 | regulation of neuronal synaptic plasticity |
| GO:0050792 | 0.003317349 | 26.91097308 | 0.086830238 | 2 | 16 | regulation of viral reproduction |
| GO:0065009 | 0.003407602 | 2.871126761 | 4.298096767 | 11 | 792 | regulation of molecular function |
| GO:0019058 | 0.00345568 | 10.78718091 | 0.303905832 | 3 | 56 | viral infectious cycle |
| GO:0051098 | 0.003610306 | 6.876332623 | 0.629519223 | 4 | 116 | regulation of binding |
| GO:0009617 | 0.003722989 | 6.81495179 | 0.634946113 | 4 | 117 | response to bacterium |
| GO:0002367 | 0.003746513 | 25.11497585 | 0.092257128 | 2 | 17 | cytokine production during immune response |
| GO:0045727 | 0.003746513 | 25.11497585 | 0.092257128 | 2 | 17 | positive regulation of translation |
| GO:0034381 | 0.004200091 | 23.54347826 | 0.097684017 | 2 | 18 | lipoprotein particle clearance |
| GO:0043066 | 0.004216422 | 4.259531773 | 1.53038294 | 6 | 282 | negative regulation of apoptosis |
| GO:0032386 | 0.004400845 | 9.853448276 | 0.331040281 | 3 | 61 | regulation of intracellular transport |
| GO:0043069 | 0.004669607 | 4.166939444 | 1.562944279 | 6 | 288 | negative regulation of programmed cell death |
| GO:0060548 | 0.004669607 | 4.166939444 | 1.562944279 | 6 | 288 | negative regulation of cell death |
| GO:0045428 | 0.004677814 | 22.15686275 | 0.103110907 | 2 | 19 | regulation of nitric oxide biosynthetic process |
| GO:0022415 | 0.005036026 | 9.366682739 | 0.347320951 | 3 | 64 | viral reproductive process |
| GO:0050804 | 0.005036026 | 9.366682739 | 0.347320951 | 3 | 64 | regulation of synaptic transmission |
| GO:0008285 | 0.005073976 | 4.092736532 | 1.590078728 | 6 | 293 | negative regulation of cell proliferation |
| GO:0016477 | 0.005242744 | 4.063774288 | 1.600932508 | 6 | 295 | cell migration |
| GO:0001315 | 0.00542689 | Inf | 0.00542689 | 1 | 1 | age-dependent response to reactive oxygen species |
| GO:0002315 | 0.00542689 | Inf | 0.00542689 | 1 | 1 | marginal zone B cell differentiation |
| GO:0002439 | 0.00542689 | Inf | 0.00542689 | 1 | 1 | chronic inflammatory response to antigenic stimulus |
| GO:0002457 | 0.00542689 | Inf | 0.00542689 | 1 | 1 | T cell antigen processing and presentation |
| GO:0002691 | 0.00542689 | Inf | 0.00542689 | 1 | 1 | regulation of cellular extravasation |
| GO:0002693 | 0.00542689 | Inf | 0.00542689 | 1 | 1 | positive regulation of cellular extravasation |
| GO:0003068 | 0.00542689 | Inf | 0.00542689 | 1 | 1 | regulation of systemic arterial blood pressure by acetylcholine |
| GO:0003069 | 0.00542689 | Inf | 0.00542689 | 1 | 1 | vasodilation by acetylcholine involved in regulation of systemic arterial blood pressure |
| GO:0003070 | 0.00542689 | Inf | 0.00542689 | 1 | 1 | regulation of systemic arterial blood pressure by neurotransmitter |
| GO:0010232 | 0.00542689 | Inf | 0.00542689 | 1 | 1 | vascular transport |
| GO:0010260 | 0.00542689 | Inf | 0.00542689 | 1 | 1 | organ senescence |
| GO:0021594 | 0.00542689 | Inf | 0.00542689 | 1 | 1 | rhombomere formation |
| GO:0021660 | 0.00542689 | Inf | 0.00542689 | 1 | 1 | rhombomere 3 formation |
| GO:0021664 | 0.00542689 | Inf | 0.00542689 | 1 | 1 | rhombomere 5 morphogenesis |
| GO:0021666 | 0.00542689 | Inf | 0.00542689 | 1 | 1 | rhombomere 5 formation |
| GO:0033004 | 0.00542689 | Inf | 0.00542689 | 1 | 1 | negative regulation of mast cell activation |
| GO:0034116 | 0.00542689 | Inf | 0.00542689 | 1 | 1 | positive regulation of heterotypic cell-cell adhesion |
| GO:0043301 | 0.00542689 | Inf | 0.00542689 | 1 | 1 | negative regulation of leukocyte degranulation |
| GO:0043305 | 0.00542689 | Inf | 0.00542689 | 1 | 1 | negative regulation of mast cell degranulation |
| GO:0045746 | 0.00542689 | Inf | 0.00542689 | 1 | 1 | negative regulation of Notch signaling pathway |
| GO:0045920 | 0.00542689 | Inf | 0.00542689 | 1 | 1 | negative regulation of exocytosis |
| GO:0045994 | 0.00542689 | Inf | 0.00542689 | 1 | 1 | positive regulation of translational initiation by iron |
| GO:0048773 | 0.00542689 | Inf | 0.00542689 | 1 | 1 | erythrophore differentiation |
| GO:0055093 | 0.00542689 | Inf | 0.00542689 | 1 | 1 | response to hyperoxia |
| GO:0070189 | 0.00542689 | Inf | 0.00542689 | 1 | 1 | kynurenine metabolic process |
| GO:0070423 | 0.00542689 | Inf | 0.00542689 | 1 | 1 | nucleotide-binding oligomerization domain containing signaling pathway |
| GO:0070427 | 0.00542689 | Inf | 0.00542689 | 1 | 1 | nucleotide-binding oligomerization domain containing 1 signaling pathway |
| GO:0070431 | 0.00542689 | Inf | 0.00542689 | 1 | 1 | nucleotide-binding oligomerization domain containing 2 signaling pathway |
| GO:0002682 | 0.005592635 | 4.007035967 | 1.622640067 | 6 | 299 | regulation of immune system process |
| GO:0048518 | 0.005747691 | 2.259838275 | 8.140334786 | 16 | 1500 | positive regulation of biological process |
| GO:0002697 | 0.00646528 | 8.523924495 | 0.37988229 | 3 | 70 | regulation of immune effector process |
| GO:0016032 | 0.00646528 | 8.523924495 | 0.37988229 | 3 | 70 | viral reproduction |
| GO:0051969 | 0.00646528 | 8.523924495 | 0.37988229 | 3 | 70 | regulation of transmission of nerve impulse |
| GO:0030099 | 0.006504304 | 5.781169341 | 0.74348391 | 4 | 137 | myeloid cell differentiation |
| GO:0046649 | 0.006553494 | 4.552212263 | 1.183061989 | 5 | 218 | lymphocyte activation |
| GO:0006366 | 0.006603237 | 2.742216465 | 4.037606054 | 10 | 744 | transcription from RNA polymerase II promoter |
| GO:0001817 | 0.006841435 | 5.694637922 | 0.75433769 | 4 | 139 | regulation of cytokine production |
| GO:0045637 | 0.006989966 | 8.275575448 | 0.39073607 | 3 | 72 | regulation of myeloid cell differentiation |
| GO:0042221 | 0.007232751 | 2.574508671 | 4.753955515 | 11 | 876 | response to chemical stimulus |
| GO:0031644 | 0.007261525 | 8.156722689 | 0.39616296 | 3 | 73 | regulation of neurological system process |
| GO:0007626 | 0.007330904 | 4.425418569 | 1.215623328 | 5 | 224 | locomotory behavior |
| GO:0050793 | 0.007356047 | 2.464461454 | 5.448597416 | 12 | 1004 | regulation of developmental process |
| GO:0007159 | 0.007419299 | 17.11462451 | 0.130245357 | 2 | 24 | leukocyte adhesion |
| GO:0008630 | 0.007419299 | 17.11462451 | 0.130245357 | 2 | 24 | DNA damage response, signal transduction resulting in induction of apoptosis |
| GO:0030217 | 0.007539275 | 8.041217896 | 0.401589849 | 3 | 74 | T cell differentiation |
| GO:0006917 | 0.007603791 | 4.384683944 | 1.226477108 | 5 | 226 | induction of apoptosis |
| GO:0012502 | 0.007742864 | 4.364591865 | 1.231903998 | 5 | 227 | induction of programmed cell death |
| GO:0001818 | 0.008036338 | 16.36925016 | 0.135672246 | 2 | 25 | negative regulation of cytokine production |
| GO:0010743 | 0.008036338 | 16.36925016 | 0.135672246 | 2 | 25 | regulation of foam cell differentiation |
| GO:0010646 | 0.008411042 | 2.639107301 | 4.18413208 | 10 | 771 | regulation of cell communication |
| GO:0048870 | 0.008539958 | 3.64946082 | 1.774592983 | 6 | 327 | cell motility |
| GO:0008637 | 0.008675692 | 15.68599034 | 0.141099136 | 2 | 26 | apoptotic mitochondrial changes |
| GO:0045732 | 0.008675692 | 15.68599034 | 0.141099136 | 2 | 26 | positive regulation of protein catabolic process |
| GO:0051235 | 0.009021991 | 7.509287926 | 0.428724299 | 3 | 79 | maintenance of location |
| GO:0042110 | 0.009109127 | 5.224895929 | 0.819460368 | 4 | 151 | T cell activation |
| GO:0019079 | 0.009337105 | 15.0573913 | 0.146526026 | 2 | 27 | viral genome replication |
| GO:0042742 | 0.009337536 | 7.411191749 | 0.434151189 | 3 | 80 | defense response to bacterium |
| GO:0051239 | 0.009446218 | 2.738561931 | 3.603454865 | 9 | 664 | regulation of multicellular organismal process |
| GO:0048583 | 0.009555977 | 3.558475567 | 1.818008102 | 6 | 335 | regulation of response to stimulus |
| GO:0009967 | 0.009880535 | 4.101181305 | 1.307880456 | 5 | 241 | positive regulation of signal transduction |

GO annotations that are significantly enriched in the set of up-regulated genes are reported. The most enriched terms are related to immune response.
