# Supplementary material for: siRNA Off-Target Effects Can Be Reduced at Concentrations That Match Their Individual Potency
Source: PLoS One. 2011 Jul 5;6(7):e21503. doi: 10.1371/journal.pone.0021503 (PMC3130022; doi:10.1371/journal.pone.0021503)
Supplement: Table S3 — HK2-3581 off-targets that are involved in immune response. (DOC) [file pone.0021503.s016.doc]

**Table S3.** HK2-3581 off-targets that are involved in immune response.

| geneSymbol | entrezGeneId | 25nMLog_2_Fc | 10nMLog_2_Fc | 1nMLog_2_Fc |
| --- | --- | --- | --- | --- |
| EXO1 | 9156 | 1.459686474 | 0.209838453 | -0.227593548 |
| SQSTM1 | 8878 | 1.265816925 | 0.061534425 | 0.033873063 |
| HMOX1 | 3162 | 1.197153641 | 0.245162135 | 0.264110692 |
| CD1D | 912 | 1.006546183 | 0.299521024 | 0.133999651 |
| SERPING1 | 710 | -1.07301775 | -0.190114159 | 0.13205329 |
| MYD88 | 4615 | -1.081173505 | -0.751814758 | -0.20963786 |
| RELB | 5971 | -1.09183379 | -0.281135365 | 0.234538653 |
| C4BPA | 722 | -1.098051929 | 0.005160485 | -0.078969068 |
| ATP6V1H | 51606 | -1.103172201 | -0.460864623 | -0.141527423 |
| PVRL2 | 5819 | -1.103383248 | -0.029616221 | 0.227457951 |
| CD83 | 9308 | -1.110330433 | -0.075504545 | -0.071516099 |
| ATP7A | 538 | -1.255013117 | -0.488851514 | -0.415310893 |
| HPX | 3263 | -1.286162773 | -0.017035556 | 0.18717892 |
| C1R | 715 | -1.317047061 | -0.173008759 | 0.163378901 |
| TNFSF4 | 7292 | -1.323659056 | 0.214969174 | -0.173532263 |
| IL4R | 3566 | -1.34540531 | -0.029213799 | 0.071808322 |
| PTX3 | 5806 | -1.345437741 | -0.431912813 | -0.190118719 |
| PAG1 | 55824 | -1.348437035 | -0.213825701 | -0.285690848 |
| CXCL16 | 58191 | -1.363492127 | -0.216221784 | 0.028248816 |
| TGFB1 | 7040 | -1.366096272 | -0.164891676 | 0.286955647 |
| A2M | 2 | -1.369021984 | -0.333352922 | -0.09215762 |
| IL32 | 9235 | -1.46527058 | -0.290683319 | 0.520950043 |
| LAT2 | 7462 | -1.481251399 | -0.336950289 | 0.043303598 |
| IL6ST | 3572 | -1.503856178 | -0.586942774 | -0.466206402 |
| ADORA2B | 136 | -1.509166908 | -0.304373136 | 0.052072408 |
| C6 | 729 | -1.51217357 | -0.347289027 | -0.168624162 |
| VTN | 7448 | -1.544573699 | -0.253210663 | 0.084750743 |
| BLNK | 29760 | -1.557236111 | -0.30795383 | -0.123456718 |
| POU2AF1 | 5450 | -1.570339752 | -0.1298835 | 0.115753955 |
| IL6R | 3570 | -1.582724306 | -0.365254482 | -0.321741894 |
| DDX58 | 23586 | -1.597213197 | -0.795147044 | -0.351185891 |
| C8B | 732 | -1.60185995 | -0.072927639 | 0.077758578 |
| CCL20 | 6364 | -1.610993988 | -0.110387961 | 0.219633898 |
| ICAM1 | 3383 | -1.612484232 | -0.522901942 | -0.062118755 |
| BNIP3L | 665 | -1.742777005 | -1.555282275 | -0.330313724 |
| ENPP1 | 5167 | -1.782267254 | -0.581531246 | -0.416569114 |
| CFB | 629 | -1.873663058 | -0.091159443 | -0.003505014 |
| TGFBR3 | 7049 | -2.029871925 | -0.881033583 | -0.364407276 |
| CX3CL1 | 6376 | -2.116597138 | -0.20321695 | 0.060322208 |
| CXCL10 | 3627 | -2.539247132 | -0.033827664 | 0.218764708 |
| CXCL12 | 6387 | -2.551981205 | -0.362903666 | -0.005022626 |
| LTB | 4050 | -2.901583226 | -0.096412788 | 0.486153685 |
| TGFB2 | 7042 | -3.344523596 | -1.556782872 | -0.62375061 |

All up-regulated and down-regulated off-targets annotated as immune response genes (GO:0006955) are described along with log_2_ fold-change values at each concentration. The majority of these genes are down-regulated.
