# Supplementary material for: siRNA Off-Target Effects Can Be Reduced at Concentrations That Match Their Individual Potency
Source: PLoS One. 2011 Jul 5;6(7):e21503. doi: 10.1371/journal.pone.0021503 (PMC3130022; doi:10.1371/journal.pone.0021503)
Supplement: Table S4 — Enrichment of HK2-3581 off-targets that are involved in cell cycle. (DOC) [file pone.0021503.s017.doc]

**Table S4.** Enrichment of HK2-3581 off-targets that are involved in cell cycle.

| GOBPID | Pvalue | OddsRatio | ExpCount | Count | Size | Term |
| --- | --- | --- | --- | --- | --- | --- |
| GO:0048285 | 1.32E-15 | 7.00020887 | 5.650997478 | 32 | 244 | organelle fission |
| GO:0000280 | 4.06E-15 | 6.956631925 | 5.488878698 | 31 | 237 | nuclear division |
| GO:0007067 | 4.06E-15 | 6.956631925 | 5.488878698 | 31 | 237 | mitosis |
| GO:0000087 | 5.15E-15 | 6.888645362 | 5.535198349 | 31 | 239 | M phase of mitotic cell cycle |
| GO:0000279 | 2.27E-13 | 5.330754546 | 7.64274249 | 34 | 330 | M phase |
| GO:0051301 | 2.41E-11 | 5.32 | 6.206833295 | 28 | 268 | cell division |
| GO:0022403 | 2.47E-11 | 4.308312163 | 9.518688374 | 35 | 411 | cell cycle phase |
| GO:0000278 | 1.30E-10 | 4.124437488 | 9.588167851 | 34 | 414 | mitotic cell cycle |
| GO:0007049 | 2.24E-10 | 3.086756066 | 18.96789727 | 50 | 819 | cell cycle |
| GO:0022402 | 2.53E-10 | 3.613138356 | 12.52946572 | 39 | 541 | cell cycle process |
| GO:0044237 | 2.44E-09 | 2.044370117 | 157.880532 | 208 | 6817 | cellular metabolic process |
| GO:0006259 | 2.93E-08 | 3.260012625 | 11.83467095 | 34 | 511 | DNA metabolic process |
| GO:0006996 | 1.90E-07 | 2.314483149 | 28.25498739 | 57 | 1220 | organelle organization |
| GO:0008152 | 3.01E-07 | 1.88515179 | 179.280211 | 221 | 7741 | metabolic process |
| GO:0006260 | 5.25E-07 | 4.340315086 | 4.93304288 | 19 | 213 | DNA replication |
| GO:0006119 | 7.20E-07 | 6.98556701 | 2.014904838 | 12 | 87 | oxidative phosphorylation |
| GO:0034960 | 2.05E-06 | 1.721589272 | 123.187113 | 163 | 5319 | cellular biopolymer metabolic process |
| GO:0007059 | 2.56E-06 | 7.754753779 | 1.528548498 | 10 | 66 | chromosome segregation |
| GO:0044260 | 3.61E-06 | 1.697731891 | 125.1556982 | 164 | 5404 | cellular macromolecule metabolic process |
| GO:0006807 | 1.69E-05 | 1.6543951 | 93.7046549 | 128 | 4046 | nitrogen compound metabolic process |
| GO:0043283 | 1.91E-05 | 1.626897584 | 134.1648704 | 170 | 5793 | biopolymer metabolic process |
| GO:0006139 | 2.89E-05 | 1.644235949 | 85.45975694 | 118 | 3690 | nucleobase, nucleoside, nucleotide and nucleic acid metabolic process |
| GO:0006091 | 3.17E-05 | 3.076298002 | 7.110066499 | 20 | 307 | generation of precursor metabolites and energy |
| GO:0006412 | 4.93E-05 | 2.805193473 | 8.545975694 | 22 | 369 | translation |
| GO:0034984 | 4.98E-05 | 2.970339437 | 7.341664756 | 20 | 317 | cellular response to DNA damage stimulus |
| GO:0006974 | 5.67E-05 | 2.853846154 | 8.013299702 | 21 | 346 | response to DNA damage stimulus |
| GO:0043170 | 6.41E-05 | 1.573146516 | 136.619812 | 170 | 5899 | macromolecule metabolic process |
| GO:0044238 | 0.000106999 | 1.565191581 | 161.9798211 | 194 | 6994 | primary metabolic process |
| GO:0016043 | 0.000120064 | 1.694126437 | 49.86310479 | 75 | 2153 | cellular component organization |
| GO:0022900 | 0.000134797 | 4.655950677 | 2.38546205 | 10 | 103 | electron transport chain |
| GO:0045333 | 0.00016397 | 5.050225285 | 1.991745013 | 9 | 86 | cellular respiration |
| GO:0006396 | 0.000185561 | 2.339335535 | 12.01994955 | 26 | 519 | RNA processing |
| GO:0006120 | 0.0001864 | 8.308243728 | 0.856913552 | 6 | 37 | mitochondrial electron transport, NADH to ubiquinone |
| GO:0006281 | 0.000187193 | 2.943129993 | 6.253152947 | 17 | 270 | DNA repair |
| GO:0015992 | 0.000405377 | 5.788461538 | 1.366429718 | 7 | 59 | proton transport |
| GO:0000070 | 0.000408925 | 9.306244529 | 0.64847512 | 5 | 28 | mitotic sister chromatid segregation |
| GO:0007051 | 0.000408925 | 9.306244529 | 0.64847512 | 5 | 28 | spindle organization |
| GO:0000226 | 0.000410635 | 4.004550626 | 2.732859436 | 10 | 118 | microtubule cytoskeleton organization |
| GO:0033554 | 0.000429202 | 2.283277716 | 11.30199496 | 24 | 488 | cellular response to stress |
| GO:0000819 | 0.000484847 | 8.917785235 | 0.671634946 | 5 | 29 | sister chromatid segregation |
| GO:0006818 | 0.000498377 | 5.573198198 | 1.412749369 | 7 | 61 | hydrogen transport |
| GO:0007017 | 0.00056239 | 2.986362711 | 5.048842009 | 14 | 218 | microtubule-based process |
| GO:0042773 | 0.00063072 | 6.434343434 | 1.065351983 | 6 | 46 | ATP synthesis coupled electron transport |
| GO:0042775 | 0.00063072 | 6.434343434 | 1.065351983 | 6 | 46 | mitochondrial ATP synthesis coupled electron transport |
| GO:0008380 | 0.000801398 | 2.653276984 | 6.461591378 | 16 | 279 | RNA splicing |
| GO:0055114 | 0.00102105 | 2.067518782 | 13.45585875 | 26 | 581 | oxidation reduction |
| GO:0022904 | 0.001220691 | 5.592446201 | 1.204310938 | 6 | 52 | respiratory electron transport chain |
| GO:0044249 | 0.001496763 | 1.445548712 | 90.67071773 | 115 | 3915 | cellular biosynthetic process |
| GO:0015985 | 0.001529992 | 6.684144295 | 0.856913552 | 5 | 37 | energy coupled proton transport, down electrochemical gradient |
| GO:0015986 | 0.001529992 | 6.684144295 | 0.856913552 | 5 | 37 | ATP synthesis coupled proton transport |
| GO:0015980 | 0.00154865 | 3.321081649 | 3.242375602 | 10 | 140 | energy derivation by oxidation of organic compounds |
| GO:0048552 | 0.001579339 | 84.910299 | 0.069479477 | 2 | 3 | regulation of metalloenzyme activity |
| GO:0048554 | 0.001579339 | 84.910299 | 0.069479477 | 2 | 3 | positive regulation of metalloenzyme activity |
| GO:0022613 | 0.001763146 | 3.048472076 | 3.867690897 | 11 | 167 | ribonucleoprotein complex biogenesis |
| GO:0008630 | 0.002078155 | 8.535117057 | 0.555835817 | 4 | 24 | DNA damage response, signal transduction resulting in induction of apoptosis |
| GO:0009058 | 0.002332355 | 1.419746189 | 92.68562256 | 116 | 4002 | biosynthetic process |
| GO:0007018 | 0.002674866 | 3.621052632 | 2.38546205 | 8 | 103 | microtubule-based movement |
| GO:0051297 | 0.002819449 | 7.757981149 | 0.602155469 | 4 | 26 | centrosome organization |
| GO:0007080 | 0.003110315 | 42.45182724 | 0.092639303 | 2 | 4 | mitotic metaphase plate congression |
| GO:0009186 | 0.003110315 | 42.45182724 | 0.092639303 | 2 | 4 | deoxyribonucleoside diphosphate metabolic process |
| GO:0007005 | 0.003197213 | 3.509373919 | 2.454941527 | 8 | 106 | mitochondrion organization |
| GO:0000387 | 0.003250252 | 7.420095972 | 0.625315295 | 4 | 27 | spliceosomal snRNP biogenesis |
| GO:0000375 | 0.003271622 | 2.97399082 | 3.589772988 | 10 | 155 | RNA splicing, via transesterification reactions |
| GO:0000377 | 0.003271622 | 2.97399082 | 3.589772988 | 10 | 155 | RNA splicing, via transesterification reactions with bulged adenosine as nucleophile |
| GO:0000398 | 0.003271622 | 2.97399082 | 3.589772988 | 10 | 155 | nuclear mRNA splicing, via spliceosome |
| GO:0006364 | 0.00329521 | 3.901412776 | 1.945425361 | 7 | 84 | rRNA processing |
| GO:0034220 | 0.003341531 | 5.481414559 | 1.019032332 | 5 | 44 | ion transmembrane transport |
| GO:0051437 | 0.003567834 | 4.431208638 | 1.482228847 | 6 | 64 | positive regulation of ubiquitin-protein ligase activity during mitotic cell cycle |
| GO:0001522 | 0.003704002 | 11.60818182 | 0.32423756 | 3 | 14 | pseudouridine synthesis |
| GO:0051443 | 0.004160873 | 4.282828283 | 1.528548498 | 6 | 66 | positive regulation of ubiquitin-protein ligase activity |
| GO:0031023 | 0.00424204 | 6.82541806 | 0.671634946 | 4 | 29 | microtubule organizing center organization |
| GO:0016072 | 0.004271615 | 3.707582583 | 2.038064664 | 7 | 88 | rRNA metabolic process |
| GO:0010467 | 0.00439222 | 1.403274343 | 78.34969044 | 99 | 3383 | gene expression |
| GO:0051439 | 0.004483304 | 4.212286802 | 1.551708324 | 6 | 67 | regulation of ubiquitin-protein ligase activity during mitotic cell cycle |
| GO:0000080 | 0.004551348 | 10.64 | 0.347397386 | 3 | 15 | G1 phase of mitotic cell cycle |
| GO:0006930 | 0.005104638 | 28.29900332 | 0.115799129 | 2 | 5 | substrate-bound cell migration, cell extension |
| GO:0051299 | 0.005104638 | 28.29900332 | 0.115799129 | 2 | 5 | centrosome separation |
| GO:0051310 | 0.005104638 | 28.29900332 | 0.115799129 | 2 | 5 | metaphase plate congression |
| GO:0051351 | 0.005182906 | 4.077922078 | 1.598027975 | 6 | 69 | positive regulation of ligase activity |
| GO:0042312 | 0.00550661 | 9.820769231 | 0.370557212 | 3 | 16 | regulation of vasodilation |
| GO:0006397 | 0.005665395 | 2.211947279 | 7.15638615 | 15 | 309 | mRNA processing |
| GO:0051186 | 0.006011435 | 2.709124863 | 3.914010548 | 10 | 169 | cofactor metabolic process |
| GO:0010498 | 0.00648744 | 3.410780098 | 2.200183444 | 7 | 95 | proteasomal protein catabolic process |
| GO:0043161 | 0.00648744 | 3.410780098 | 2.200183444 | 7 | 95 | proteasomal ubiquitin-dependent protein catabolic process |
| GO:0009987 | 0.006517795 | 1.567222164 | 253.2758542 | 269 | 10936 | cellular process |
| GO:0006297 | 0.006573274 | 9.118571429 | 0.393717037 | 3 | 17 | nucleotide-excision repair, DNA gap filling |
| GO:0009260 | 0.006756915 | 3.067312349 | 2.779179087 | 8 | 120 | ribonucleotide biosynthetic process |
| GO:0051438 | 0.007278454 | 3.776589424 | 1.713827104 | 6 | 74 | regulation of ubiquitin-protein ligase activity |
| GO:0006449 | 0.007540166 | 21.22259136 | 0.138958954 | 2 | 6 | regulation of translational termination |
| GO:0009263 | 0.007540166 | 21.22259136 | 0.138958954 | 2 | 6 | deoxyribonucleotide biosynthetic process |
| GO:0045039 | 0.007540166 | 21.22259136 | 0.138958954 | 2 | 6 | protein import into mitochondrial inner membrane |
| GO:0048147 | 0.007540166 | 21.22259136 | 0.138958954 | 2 | 6 | negative regulation of fibroblast proliferation |
| GO:0051318 | 0.007754402 | 8.51 | 0.416876863 | 3 | 18 | G1 phase |
| GO:0016071 | 0.007816175 | 2.071038536 | 8.129098831 | 16 | 351 | mRNA metabolic process |
| GO:0051716 | 0.008352783 | 1.760765301 | 14.96124742 | 25 | 646 | cellular response to stimulus |
| GO:0051340 | 0.008796582 | 3.616161616 | 1.783306581 | 6 | 77 | regulation of ligase activity |
| GO:0034961 | 0.008931774 | 1.37590225 | 69.0394405 | 87 | 2981 | cellular biopolymer biosynthetic process |
| GO:0030307 | 0.009052661 | 7.9775 | 0.440036689 | 3 | 19 | positive regulation of cell growth |
| GO:0043284 | 0.009760819 | 1.369777319 | 69.27103875 | 87 | 2991 | biopolymer biosynthetic process |
| GO:0006310 | 0.009958255 | 3.124577703 | 2.38546205 | 7 | 103 | DNA recombination |

GO annotations that are significantly enriched in the set of up-regulated genes are reported. The most enriched terms are related to cell cycle.
