# Supplementary material for: siRNA Off-Target Effects Can Be Reduced at Concentrations That Match Their Individual Potency
Source: PLoS One. 2011 Jul 5;6(7):e21503. doi: 10.1371/journal.pone.0021503 (PMC3130022; doi:10.1371/journal.pone.0021503)
Supplement: Table S5 — HK2-3581 off-targets that are involved in cell cycle. (DOC) [file pone.0021503.s018.doc]

**Table S5.** HK2-3581 off-targets that are involved in cell cycle.

| geneSymbol | entrezGeneId | 25nMLogFc | 10nMLogFc | 1nMLogFc |
| --- | --- | --- | --- | --- |
| CDK2 | 1017 | 2.226720135 | 0.320729289 | 0.037260219 |
| RPS27L | 51065 | 1.935408358 | 0.499977779 | 0.34779404 |
| PBK | 55872 | 1.9076518 | 0.489607356 | -0.0175936 |
| NDC80 | 10403 | 1.848865821 | 0.414809418 | -0.146620848 |
| UBE2C | 11065 | 1.763513767 | 1.122745787 | 0.784476725 |
| MND1 | 84057 | 1.616295728 | 1.034668976 | 0.54357134 |
| E2F7 | 144455 | 1.592127632 | 0.092523058 | -0.234824883 |
| C11orf82 | 220042 | 1.590089295 | 0.201699822 | -0.290840815 |
| NCAPG | 64151 | 1.551746439 | 0.238723444 | -0.2572938 |
| CDK6 | 1021 | 1.546580972 | -0.142989832 | -0.205107114 |
| PTTG1 | 9232 | 1.545433129 | 1.043450852 | 0.700378457 |
| EXO1 | 9156 | 1.459686474 | 0.209838453 | -0.227593548 |
| OIP5 | 11339 | 1.457399816 | 0.588706405 | 0.322570363 |
| CDCA5 | 113130 | 1.408962816 | 0.436969571 | 0.153497675 |
| FANCD2 | 2177 | 1.400918551 | 0.584399307 | 0.286556065 |
| AURKA | 6790 | 1.363914028 | 0.300060907 | -0.008833219 |
| PLK1 | 5347 | 1.334499244 | 0.24499349 | 0.210188595 |
| AURKB | 9212 | 1.269732901 | 0.543298941 | 0.435681012 |
| NUF2 | 83540 | 1.254019772 | 0.08617938 | -0.327043945 |
| PA2G4 | 5036 | 1.245214016 | 0.519616279 | 0.151989685 |
| KIF23 | 9493 | 1.242911995 | 0.04179853 | -0.292570238 |
| MNS1 | 55329 | 1.233396714 | 0.024328834 | -0.322916113 |
| CDC20 | 991 | 1.229241849 | 0.402574939 | 0.260417214 |
| KIF2C | 11004 | 1.222332737 | 0.323970882 | 0.03106123 |
| ERCC6L | 54821 | 1.198552248 | 0.173678608 | -0.054257349 |
| KIF15 | 56992 | 1.190417674 | 0.178281456 | -0.144728036 |
| MRPL41 | 64975 | 1.188959678 | 1.208322196 | 1.058837263 |
| BRCA1 | 672 | 1.170101435 | 0.220641462 | -0.000505142 |
| NEK2 | 4751 | 1.155880977 | 0.006399234 | -0.276601584 |
| FANCI | 55215 | 1.143647695 | 0.224219858 | -0.158072394 |
| HAUS8 | 93323 | 1.143388454 | 0.323213139 | 0.156772443 |
| CDC2 | 983 | 1.133827681 | 0.320848527 | -0.119989682 |
| GSG2 | 83903 | 1.13075647 | 0.330219298 | -0.003492465 |
| CCNB1 | 891 | 1.118869634 | 0.232975708 | -0.072230939 |
| SSSCA1 | 10534 | 1.117174995 | 0.983005219 | 0.869819083 |
| KIFC1 | 3833 | 1.116070955 | 0.219716762 | 0.054448017 |
| ESCO2 | 157570 | 1.11579533 | 0.07210336 | -0.173384512 |
| RACGAP1 | 29127 | 1.112655719 | 0.179444609 | -0.098816822 |
| CDCA3 | 83461 | 1.090567825 | 0.580901904 | 0.337143991 |
| SPC25 | 57405 | 1.090471932 | 0.536656478 | 0.422383946 |
| HJURP | 55355 | 1.086792673 | 0.215783217 | -0.052091277 |
| SKA1 | 220134 | 1.077996036 | -0.17906985 | -0.255006857 |
| KIF11 | 3832 | 1.052458545 | -0.076840912 | -0.291154159 |
| DTYMK | 1841 | 1.038433806 | 0.574232152 | 0.364844018 |
| TUSC2 | 11334 | 1.015886346 | 0.6806567 | 0.447908499 |
| CD2AP | 23607 | 1.008728874 | 0.081677875 | -0.082006706 |
| FANCG | 2189 | 1.006952473 | 0.128673335 | 0.080905407 |
| CDC23 | 8697 | 1.005130532 | 0.000251804 | -0.244529036 |
| SUGT1 | 10910 | 1.002649321 | 0.404500327 | 0.16800636 |
| KIF20B | 9585 | 1.001668089 | -0.180598562 | -0.297116894 |
| RASSF4 | 83937 | -1.022129516 | -0.327874164 | 0.059057458 |
| AKT1 | 207 | -1.025937362 | -0.471614508 | -0.017684916 |
| ARHGEF2 | 9181 | -1.033755508 | -0.112523399 | 0.060931353 |
| PROX1 | 5629 | -1.034808977 | -0.362697461 | -0.343225491 |
| MAP3K8 | 1326 | -1.054643953 | -0.653473053 | -0.571022259 |
| RAD50 | 10111 | -1.074568972 | -0.392346811 | -0.268290134 |
| MLH3 | 27030 | -1.088472621 | -0.116232365 | -0.067544863 |
| TEX15 | 56154 | -1.109105886 | -0.710176757 | -0.589537671 |
| MAPRE2 | 10982 | -1.1149246 | -0.399950397 | -0.240422801 |
| SPAST | 6683 | -1.118043995 | -1.060730563 | -0.715677354 |
| FSD1 | 79187 | -1.128126736 | -0.606503526 | -0.035604525 |
| CCND1 | 595 | -1.12850442 | -0.110706153 | 0.011692777 |
| RHOB | 388 | -1.144777939 | -0.17417757 | -0.003134609 |
| CCNE1 | 898 | -1.195115182 | -0.314667331 | -0.027383582 |
| BMP2 | 650 | -1.223367726 | -0.349993898 | -0.152016521 |
| TSPYL2 | 64061 | -1.281883204 | -0.146118043 | 0.285947924 |
| BCAT1 | 586 | -1.284295198 | -0.695627818 | -0.407855266 |
| PKD2 | 5311 | -1.330628937 | -0.318071757 | -0.2808007 |
| TGFB1 | 7040 | -1.366096272 | -0.164891676 | 0.286955647 |
| CUL5 | 8065 | -1.383889473 | -0.442414286 | -0.358525711 |
| PDE3A | 5139 | -1.435401871 | -0.79770188 | -0.452380654 |
| TET2 | 54790 | -1.466535806 | -0.67181227 | -0.393437695 |
| TCF7L1 | 83439 | -1.469202726 | -0.068782421 | 0.36606466 |
| NEDD9 | 4739 | -1.474939495 | -0.215714284 | -0.111151439 |
| TRNP1 | 388610 | -1.568315038 | 0.091307747 | 0.304826659 |
| CDKN2B | 1030 | -1.617180502 | -0.362021295 | -0.236305253 |
| RHOU | 58480 | -1.773530026 | -0.301639872 | -0.056346679 |
| CABLES2 | 81928 | -2.072219744 | -0.763803185 | -0.094155233 |
| PLK2 | 10769 | -2.185362321 | -0.787173873 | -0.310111472 |
| SESN3 | 143686 | -2.196116026 | -0.547268022 | -0.350993289 |
| HSPA2 | 3306 | -2.472758641 | -1.657945748 | -0.621960048 |
| LTB | 4050 | -2.901583226 | -0.096412788 | 0.486153685 |
| TGFB2 | 7042 | -3.344523596 | -1.556782872 | -0.62375061 |

All up-regulated and down-regulated off-targets annotated as cell cycle genes (GO:0007049) are described along with log_2_ fold-change values at each concentration. The majority of these genes are up-regulated.
