# Supplementary material for: siRNA Off-Target Effects Can Be Reduced at Concentrations That Match Their Individual Potency
Source: PLoS One. 2011 Jul 5;6(7):e21503. doi: 10.1371/journal.pone.0021503 (PMC3130022; doi:10.1371/journal.pone.0021503)
Supplement: Table S6 — Enrichment of HK2-3581M off-targets that are involved in cell cycle. (DOC) [file pone.0021503.s019.doc]

**Table S6. Enrichment of HK2-3581M off-targets that are involved in cell cycle.**

| GOBPID | Pvalue | OddsRatio | ExpCount | Count | Size | Term |
| --- | --- | --- | --- | --- | --- | --- |
| GO:0000279 | 1.33E-07 | 11.35535714 | 1.135060766 | 10 | 330 | M phase |
| GO:0022403 | 1.00E-06 | 9.003918774 | 1.41366659 | 10 | 411 | cell cycle phase |
| GO:0022402 | 1.54E-06 | 7.635294118 | 1.86081174 | 11 | 541 | cell cycle process |
| GO:0007049 | 1.41E-05 | 5.511321392 | 2.817014446 | 12 | 819 | cell cycle |
| GO:0000280 | 1.48E-05 | 10.25812357 | 0.815180005 | 7 | 237 | nuclear division |
| GO:0007067 | 1.48E-05 | 10.25812357 | 0.815180005 | 7 | 237 | mitosis |
| GO:0000087 | 1.56E-05 | 10.16810345 | 0.822059161 | 7 | 239 | M phase of mitotic cell cycle |
| GO:0048285 | 1.79E-05 | 9.9497002 | 0.839257051 | 7 | 244 | organelle fission |
| GO:0051301 | 0.000290972 | 7.502055197 | 0.921806925 | 6 | 268 | cell division |
| GO:0000278 | 0.000484428 | 5.716862796 | 1.423985324 | 7 | 414 | mitotic cell cycle |
| GO:0043603 | 0.000487758 | 22.10204082 | 0.154781014 | 3 | 45 | cellular amide metabolic process |
| GO:0006098 | 0.00051154 | 75.75581395 | 0.034395781 | 2 | 10 | pentose-phosphate shunt |
| GO:0006740 | 0.00051154 | 75.75581395 | 0.034395781 | 2 | 10 | NADPH regeneration |
| GO:0006739 | 0.0011806 | 46.60107335 | 0.051593671 | 2 | 15 | NADP metabolic process |
| GO:0006996 | 0.002300949 | 3.165450299 | 4.196285256 | 11 | 1220 | organelle organization |
| GO:0019321 | 0.002557876 | 30.2744186 | 0.075670718 | 2 | 22 | pentose metabolic process |
| GO:0006010 | 0.003439578 | Inf | 0.003439578 | 1 | 1 | glucose 6-phosphate utilization |
| GO:0009052 | 0.003439578 | Inf | 0.003439578 | 1 | 1 | pentose-phosphate shunt, non-oxidative branch |
| GO:0010731 | 0.003439578 | Inf | 0.003439578 | 1 | 1 | protein amino acid glutathionylation |
| GO:0010732 | 0.003439578 | Inf | 0.003439578 | 1 | 1 | regulation of protein amino acid glutathionylation |
| GO:0010734 | 0.003439578 | Inf | 0.003439578 | 1 | 1 | negative regulation of protein amino acid glutathionylation |
| GO:0019302 | 0.003439578 | Inf | 0.003439578 | 1 | 1 | D-ribose biosynthetic process |
| GO:0019693 | 0.003439578 | Inf | 0.003439578 | 1 | 1 | ribose phosphate metabolic process |
| GO:0031581 | 0.003439578 | Inf | 0.003439578 | 1 | 1 | hemidesmosome assembly |
| GO:0046390 | 0.003439578 | Inf | 0.003439578 | 1 | 1 | ribose phosphate biosynthetic process |
| GO:0070408 | 0.003439578 | Inf | 0.003439578 | 1 | 1 | carbamoyl phosphate metabolic process |
| GO:0070409 | 0.003439578 | Inf | 0.003439578 | 1 | 1 | carbamoyl phosphate biosynthetic process |
| GO:0000070 | 0.004131148 | 23.27728086 | 0.096308186 | 2 | 28 | mitotic sister chromatid segregation |
| GO:0007051 | 0.004131148 | 23.27728086 | 0.096308186 | 2 | 28 | spindle organization |
| GO:0016043 | 0.004193305 | 2.549111319 | 7.405411603 | 15 | 2153 | cellular component organization |
| GO:0000819 | 0.004427491 | 22.41343669 | 0.099747764 | 2 | 29 | sister chromatid segregation |
| GO:0006769 | 0.005048828 | 20.86447474 | 0.10662692 | 2 | 31 | nicotinamide metabolic process |
| GO:0046496 | 0.005048828 | 20.86447474 | 0.10662692 | 2 | 31 | nicotinamide nucleotide metabolic process |
| GO:0009820 | 0.00537369 | 20.16744186 | 0.110066499 | 2 | 32 | alkaloid metabolic process |
| GO:0016052 | 0.005881933 | 8.883241758 | 0.368034854 | 3 | 107 | carbohydrate catabolic process |
| GO:0006260 | 0.006038895 | 5.988563426 | 0.732630131 | 4 | 213 | DNA replication |
| GO:0019362 | 0.006051471 | 18.90406977 | 0.116945655 | 2 | 34 | pyridine nucleotide metabolic process |
| GO:0009051 | 0.006867587 | 296.2954545 | 0.006879156 | 1 | 2 | pentose-phosphate shunt, oxidative branch |
| GO:0019322 | 0.006867587 | 296.2954545 | 0.006879156 | 1 | 2 | pentose biosynthetic process |
| GO:0022027 | 0.006867587 | 296.2954545 | 0.006879156 | 1 | 2 | interkinetic nuclear migration |
| GO:0022617 | 0.006867587 | 296.2954545 | 0.006879156 | 1 | 2 | extracellular matrix disassembly |
| GO:0030953 | 0.006867587 | 296.2954545 | 0.006879156 | 1 | 2 | spindle astral microtubule organization |
| GO:0006733 | 0.009984773 | 14.39202658 | 0.151341435 | 2 | 44 | oxidoreduction coenzyme metabolic process |

GO annotations that are significantly enriched in the set of up-regulated genes are reported. The most enriched terms are related to cell cycle.
