# Supplementary material for: siRNA Off-Target Effects Can Be Reduced at Concentrations That Match Their Individual Potency
Source: PLoS One. 2011 Jul 5;6(7):e21503. doi: 10.1371/journal.pone.0021503 (PMC3130022; doi:10.1371/journal.pone.0021503)
Supplement: Table S7 — HK2-3581M off-targets that are involved in cell cycle. (DOC) [file pone.0021503.s020.doc]

**Table S7.** HK2-3581M off-targets that are involved in cell cycle.

| geneSymbol | entrezGeneId | 25nMLogFc | 10nMLogFc | 1nMLogFc |
| --- | --- | --- | --- | --- |
| PLK1 | 5347 | 1.54316083 | 0.051058744 | -0.1448069 |
| CDCA5 | 113130 | 1.328351213 | 0.149922379 | -0.066646729 |
| CDC20 | 991 | 1.236696689 | -0.036816569 | -0.144360831 |
| TACC3 | 10460 | 1.212794087 | -0.17790673 | -0.123052075 |
| CDK2 | 1017 | 1.198656356 | 0.196173389 | -0.10190347 |
| PBK | 55872 | 1.098280188 | 0.230524895 | 0.008099591 |
| MNS1 | 55329 | 1.091806346 | 0.062702128 | -0.044114582 |
| E2F7 | 144455 | 1.082791291 | 0.267144934 | 0.00374292 |
| AURKB | 9212 | 1.065078897 | -0.247557798 | -0.195182622 |
| C11orf82 | 220042 | 1.063382199 | 0.082428716 | -0.065989916 |
| NDC80 | 10403 | 1.048772767 | -0.143348441 | -0.108634179 |
| FBXO43 | 286151 | 1.001401157 | 0.308198772 | 0.080020171 |
| TXNIP | 10628 | -1.039452898 | -0.268450395 | -0.089094411 |
| PSMC3 | 5702 | -1.044724136 | -0.07256643 | -0.241453576 |
| NEDD1 | 121441 | -1.070211347 | -0.248046762 | -0.399337743 |
| SESN3 | 143686 | -1.360399989 | -0.231709377 | 0.069209924 |
| TGFB2 | 7042 | -1.596122835 | -0.49213349 | -0.223882924 |
| LTB | 4050 | -1.871566906 | -0.489548205 | -0.101330705 |

All up-regulated and down-regulated off-targets annotated as cell cycle genes (GO:0007049) are described along with log_2_ fold-change values at each concentration. The majority of these genes are up-regulated.
